# Microbiota-dependent biotransformation of apiin drives butyrate-associated protection against colitis

**DOI:** 10.1038/s41538-026-00992-4

**Published:** 2026-07-14

**Authors:** Zhenning Guo, Meiqi Zhao, Yuwei Wu, Peng Zhang, Shiming Li

**Affiliations:** 1 Tianjin Key Laboratory of Life and Health Detection, Life and Health Intelligent Research Institute, Tianjin University of Technology, Tianjin, China; 2 Gastroenterology and Hepatology Department & Department of Cardiology, Tianjin First Central Hospital, Tianjin, China; 3 Department of Food Science, Rutgers University, New Brunswick, New Jersey USA; 4 College of Biology and Agricultural Resources, Huanggang Normal University, Huanggang, China

**Keywords:** Diseases, Gastroenterology, Immunology, Microbiology

## Abstract

Dietary glycosides represent an important class of microbiota-responsive food components with potential benefits for intestinal health, yet their biotransformation and functional mechanisms remain incompletely understood. Here, we investigated the protective effects of apiin (apigenin-7-O-apiosylglucoside), a naturally occurring flavone glycoside abundant in plant-based foods, against dextran sulfate sodium (DSS)-induced colitis. We demonstrate that apiin is resistant to host digestion but undergoes efficient microbial deglycosylation in the distal intestine, yielding the bioactive aglycone apigenin. Apiin administration was associated with alleviation of colitis severity, as evidenced by improved clinical and histopathological outcomes, reduced pro-inflammatory cytokine production (TNF-α, IL-1β, IL-6), and restoration of intestinal barrier integrity. Integrative multi-omics analyses revealed that apiin reshaped gut microbiota composition and function, leading to enhanced production of the short-chain fatty acid butyrate. This metabolic shift was associated with G protein-coupled receptors (GPR41 and GPR43) and PPARγ signaling, as well as NF-κB-driven inflammatory responses. Importantly, fecal microbiota transplantation recapitulated the protective effects of apiin, confirming a microbiota-dependent mechanism. Collectively, our findings identify apiin as a microbiota-dependent pro-flavonoid whose colonic biotransformation into apigenin and butyrate-associated signaling may contribute to its anti-inflammatory activity.

## Introduction

Inflammatory bowel disease (IBD), including ulcerative colitis (UC) and Crohn’s disease (CD), is a chronic and relapsing inflammatory disorder of the gastrointestinal tract characterized by epithelial barrier disruption, dysregulated immune responses, and intestinal dysbiosis^1–3^. The rising global incidence of IBD has imposed a substantial public health burden, while existing therapeutic strategies such as corticosteroids, biologics, and immunosuppressants, are often constrained by adverse effects and limited long-term efficacy^4,5^. Consequently, increasing attention has turned to dietary bioactives derived from natural products as promising, safer, and effective alternatives for maintaining intestinal homeostasis and attenuating inflammation.

Gut microbiota is widely acknowledged as a critical factor in influencing intestinal health via regulating immune balance and maintaining barrier integrity^6,7^. Generally, IBD is characterized by gut microbial dysbiosis, typically marked by reduced abundances of *Bacteroides* and *Akkermansia*, alongside an increased prevalence of *Proteobacteria*, particularly *Escherichia*^8^. Among microbial metabolites, butyrate plays a particularly vital role in intestinal health, serving as a primary energy source for colonocytes and a potent anti-inflammatory molecule that regulates G-protein–coupled receptors (GPR41 and GPR43) and peroxisome proliferator-activated receptor gamma (PPARγ)^9–11^. Reduced butyrate levels are reported to be strongly associated with epithelial injury and mucosal inflammation, suggesting that interventions enhancing butyrate-producing bacteria or activating the signaling pathways could effectively prevent or mitigate IBD^12–14^.

Flavonoids, abundant in fruits and vegetables, exhibit significant biological activity in modulating the composition and function of gut microbiota, as well as the intestinal health^11^. Apiin (apigenin-7-O-apiosylglucoside), a naturally occurring diglycosylated flavone enriched in celery (*Apium graveolens*) and parsley (*Petroselinum crispum*), possesses antioxidant and anti-inflammatory properties^15,16^. Unlike apigenin, apiin contains an apiosylglucoside moiety that markedly reduces its absorption in the small intestine, thereby increasing its availability for microbial biotransformation in the distal gut^17^. This distinct metabolic fate suggests that apiin may exert microbiota-dependent effects that differ from those of apigenin or other flavonoid glycosides. Although several flavonoids have shown protective effects against colitis^18,19^, the mechanisms by which apiin modulates gut microbial composition and short-chain fatty acid (SCFAs) metabolism, particularly butyrate-associated signaling, remain unclear.

Herein, we hypothesized that apiin alleviates colitis by reshaping gut microbiota composition, enhancing butyrate production, and restoring butyrate-associated GPRs and PPARγ signaling, thereby strengthening intestinal barrier function and suppressing inflammation. To test this hypothesis, we employed a dextran sulfate sodium (DSS)-induced colitis mouse model integrated with metagenomic, metabolomic, and molecular analyses. We assessed the effects of apiin on clinical symptoms, histopathology, cytokine production, and intestinal barrier integrity; identified microbial taxa and enzymes associated with butyrate synthesis; and verified the causal role of the microbiota via fecal microbiota transplantation (FMT). Collectively, our study provides new insight into microbiota–metabolite–host axis through which apiin confers protective effects against intestinal inflammation, offering a scientific foundation for developing apiin-rich foods or nutraceuticals for IBD prevention.

## Results

### Structural identification and chemical characterization of apiin

**Fig. 1:**
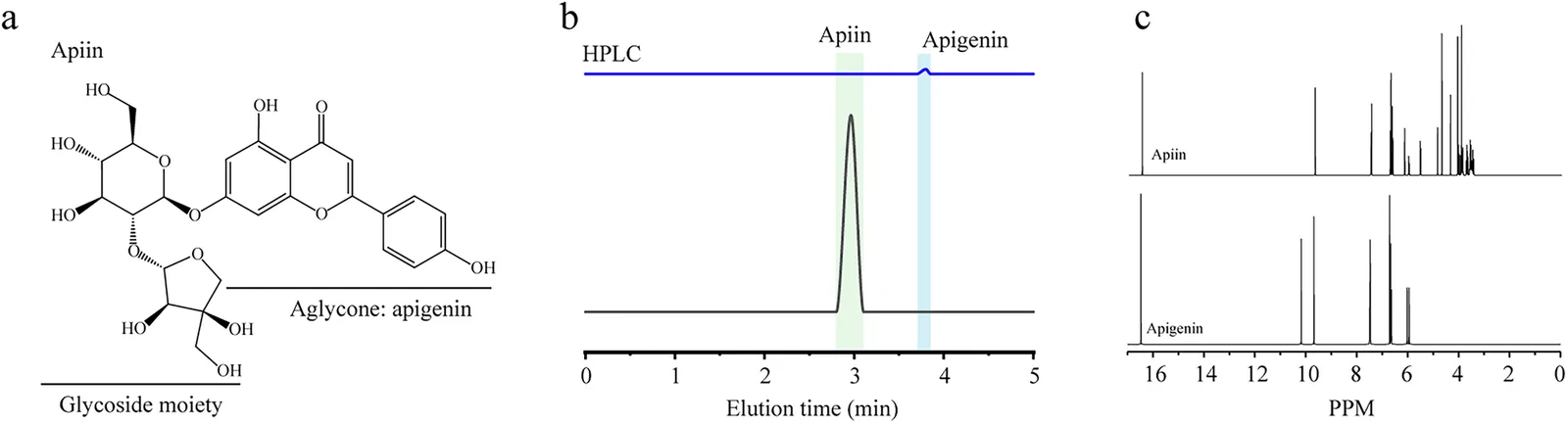
Structural and analytical characterization of apiin and apigenin. **a** Chemical structure of asiaticoside. **b** HPLC chromatograms displaying distinct retention times for apiin and apigenin. **c** ¹H-NMR spectra of apiin and apigenin.

The chemical structure of apiin (apigenin-7-O-apiosylglucoside) is presented in Fig. 1a, illustrating its flavone backbone conjugated with an apiosyl–glucosyl disaccharide moiety at the C-7 position. HPLC analysis was performed to confirm compound identity and purity, apiin and apigenin exhibited distinct chromatographic retention times under identical conditions (Fig. 1b), with no detectable co-eluting impurities. Additionally, ^1^H NMR spectra of apiin and apigenin displayed characteristic proton signals corresponding to sugar moieties in the δ 3.0–5.5 ppm region, which were absent in apigenin. In contrast, apigenin exhibited typical flavone aromatic proton signals without glycosidic proton resonances. These spectral differences further support the structural integrity of apiin and confirm its glycosylated configuration.

### Gastrointestinal distribution and biotransformation of apiin following oral administration in mice

**Fig. 2:**
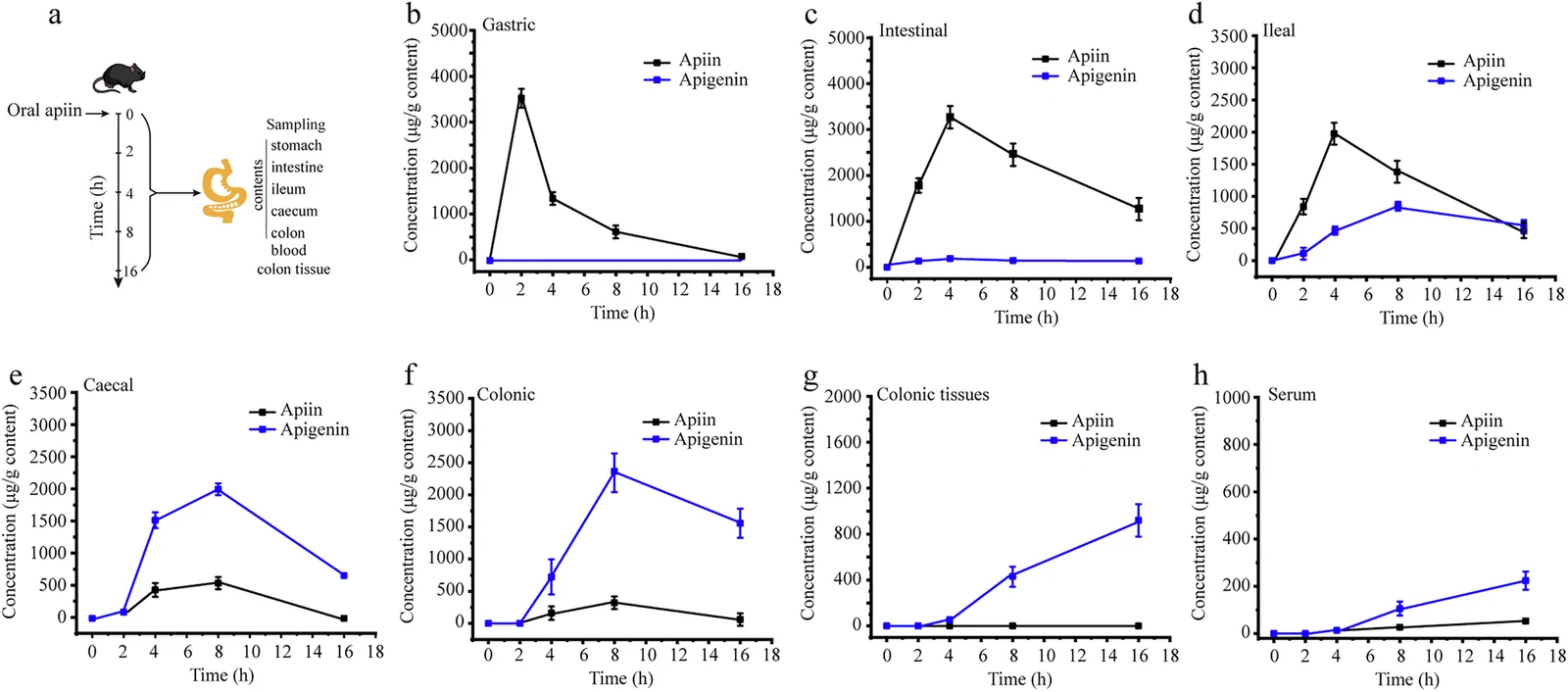
Gastrointestinal distribution and biotransformation of apiin following oral administration. **a** Experimental design showing sampling sites and time points. **b**–**f** Concentrations of apiin and apigenin in gastrointestinal contents (stomach, small intestine, ileum, cecum, and colon). **g** The concentration of apiin and apigenin in colonic tissues. **h** The concentration of apiin and apigenin in serum samples. Samples were collected at 2, 4, 8, and 16 h post-gavage.

To determine the gastrointestinal fate of apiin, concentrations of apiin and its aglycone apigenin were quantified in digestive tract segments, colonic tissues, and serum at 2, 4, 8, and 16 h post-oral gavage (Fig. 2a). In the stomach (Fig. 2b), apiin levels decreased progressively from 2 to 16 h, with no detectable apigenin. In the small intestine (Fig. 2c), apiin peaked at 4 h and then declined, while apigenin remained undetectable, indicating minimal hydrolysis of apiin in the proximal intestine. In the ileum (Fig. 2d), apiin also peaked at 4 h, whereas apigenin increased to 8 h before declining, suggesting initiation of deglycosylation in the distal small intestine. In the cecum (Fig. 2e), apiin accumulated from 2 to 8 h, and apigenin similarly peaked at 8 h, indicating active microbial biotransformation. In the colonic contents (Fig. 2f), apiin remained low, while apigenin increased to 8 h and then decreased. In colonic tissues (Fig. 2g) and serum (Fig. 2h), apiin levels were consistently low, whereas apigenin progressively increased up to 16 h. These findings indicate that apiin functions as a colon-delivered flavonoid glycoside with microbiota-mediated conversion to apigenin, supporting a gut-localized biotransformation process that may contribute to its anti-colitic effects. Both apiin and apigenin remained detectable in distal intestinal compartments, indicating that microbial deglycosylation continued throughout the cecum and colon.

### Apiin attenuated symptoms of DSS-induced colitis in mice

**Fig. 3:**
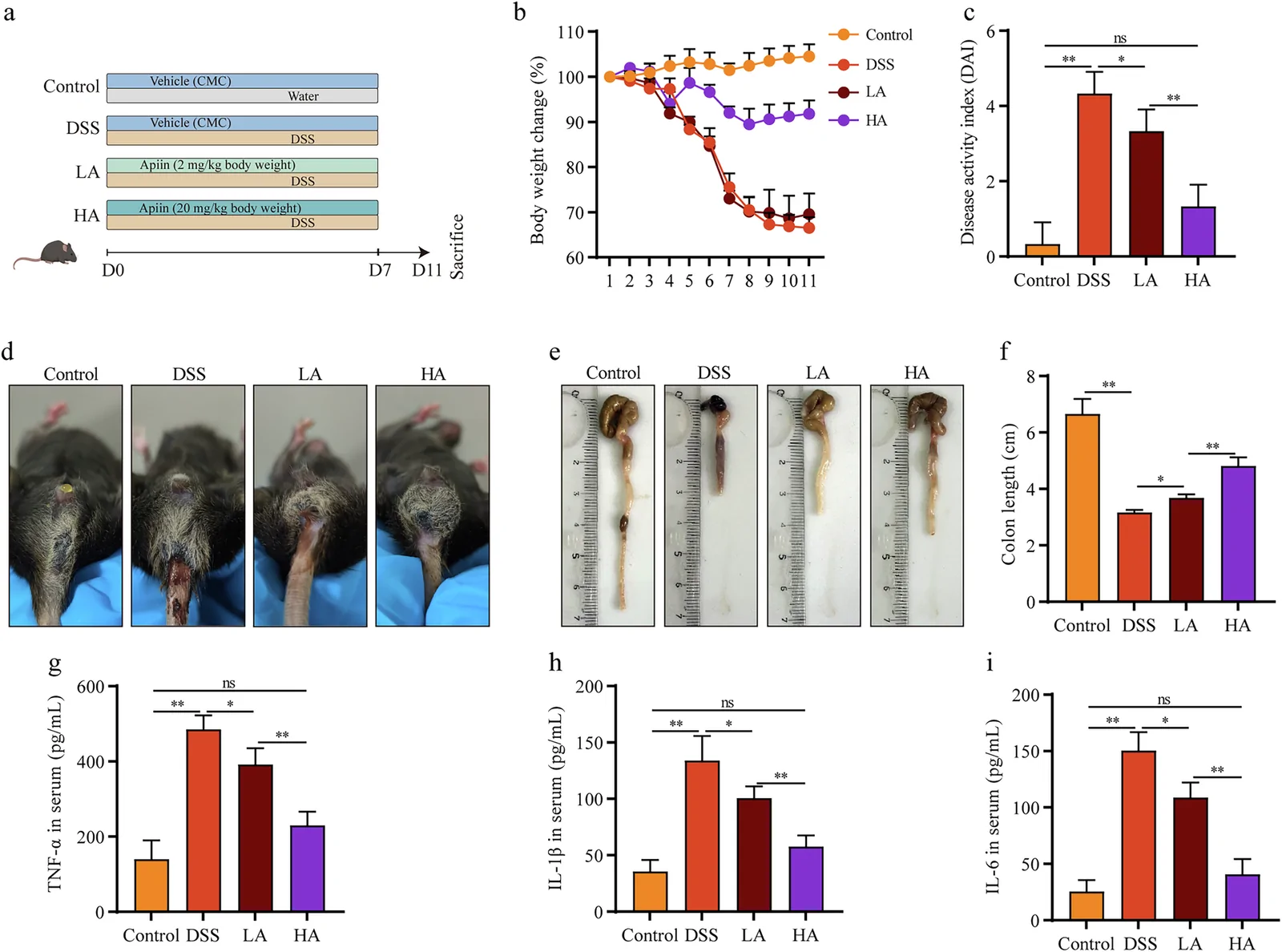
Apiin treatment attenuates symptoms of DSS-induced colitis in mice. **a** Schematic representation of the experimental design. **b** Body weight changes during the experimental period. **c** Disease activity index (DAI) scores. **d** Representative images showing fecal occult blood and hematochezia. **e** Representative images of colon morphology. **f** Statistical analysis of colon length. **g**–**i** Serum concentrations of TNF-α, IL-1β, and IL-6, respectively. *p < 0.05, **p < 0.01; ns, not significant.

The protective effects of apiin were evaluated in a DSS-induced colitis mouse model at different concentrations (2 and 20 mg/kg body weight/day) (Fig. 3a). As a result, DSS treatment caused significant body weight loss, increased disease activity index (DAI) scores, hematochezia, and reduction in colon length compared with the control group (*p* < 0.01; Fig. 3b–f). Low-dose apiin (2 mg/kg) did not affect body weight but led to modest improvements in DAI scores, hematochezia, and colon length (*p* < 0.05). In contrast, high-dose apiin (20 mg/kg) markedly alleviated DSS-induced colitis, restoring DAI scores, hematochezia, and colon length to levels comparable to controls (*p* < 0.01). To further assess the protective effects of apiin, serum levels of the pro-inflammatory cytokines including TNF-α, IL-1β, and IL-6, were measured. The levels of these cytokines were significantly elevated in DSS-treated mice (*p* < 0.01; Fig. 3g–i), while the treatment of high-dose apiin significantly reduced all three cytokines to levels similar to those in the control group. Collectively, these findings demonstrate that apiin, particularly at a high dose, effectively attenuated DSS-induced colitis symptoms in mice.

### Apiin treatment participated in modulating gut microbiota composition in colitis mice

**Fig. 4:**
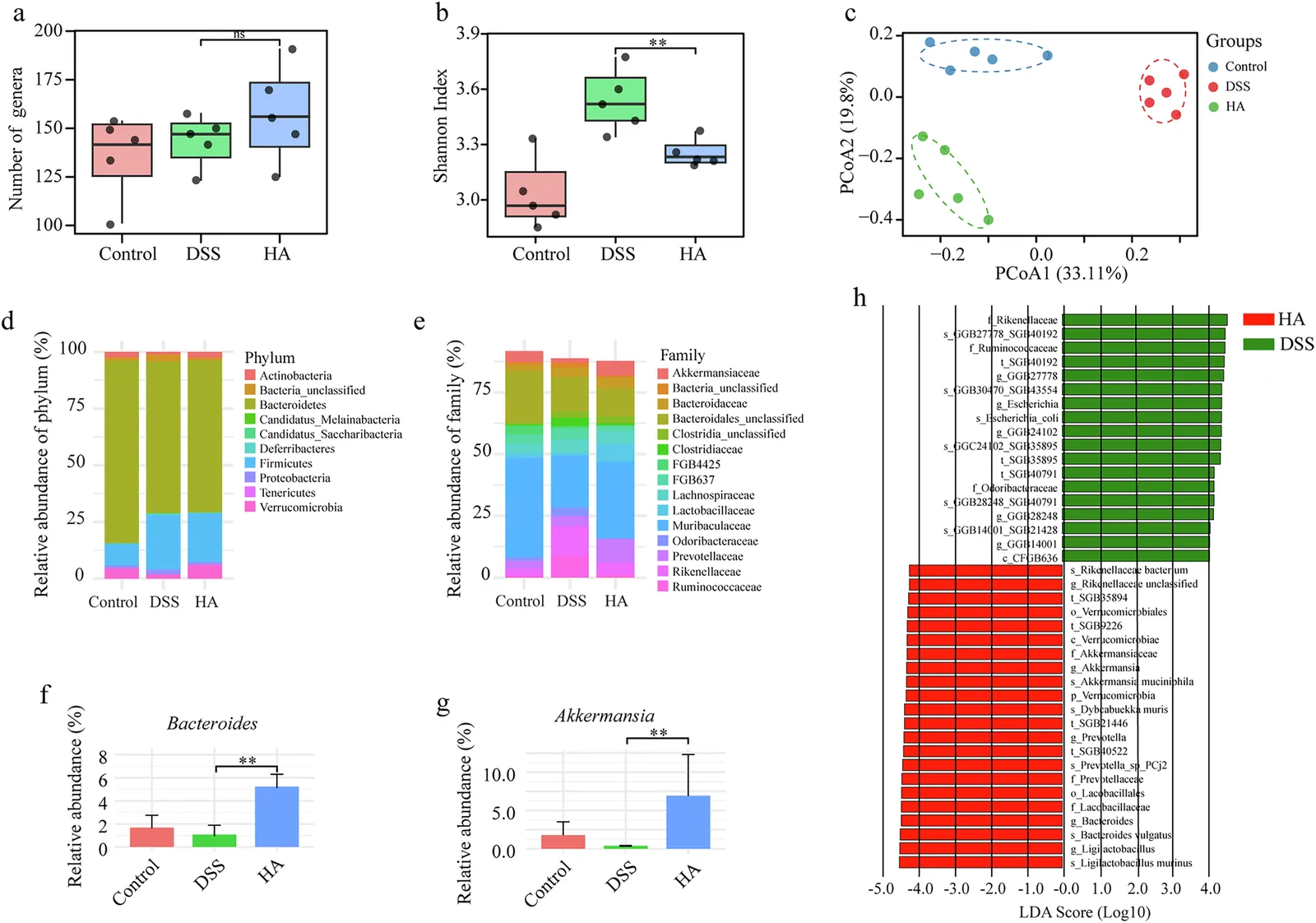
Apiin treatment modulates gut microbiota composition and diversity in DSS-induced colitis mice. **a** Number of bacterial genera detected in each group. **b** α-Diversity analysis of gut microbiota based on the Shannon index. **c** Principal coordinate analysis (PCoA) of β-diversity using the Bray–Curtis distance metric. **d** Relative abundance of the top 10 bacterial phyla. **e** Relative abundance of the top 15 bacterial families. **f** LEfSe analysis identifying differentially enriched taxa among groups (LDA > 4.0). **g**, **h** Relative abundances of Bacteroides and Ligilactobacillus across treatment groups. *p < 0.05, **p < 0.01; ns, not significant.

The gut microbiota plays a central role in maintaining intestinal homeostasis, and intestinal dysbiosis is a key pathological feature of colitis^20^. To investigate the impact of apiin on gut microbial communities in DSS-induced colitis, metagenomic sequencing was performed. Differences among the Control, DSS, and high-dose apiin (HA) groups, were evaluated using genus richness and Shannon diversity indices (Fig. 4a,b). β-Diversity analysis based on the Bray–Curtis matrix revealed a distinct separation between the Control and DSS groups, whereas the microbial community structure of the HA group shifted toward that of the Control, indicating partial restoration via apiin treatment (Fig. 4c). At the phylum level, DSS treatment increased the relative abundance of Proteobacteria and Firmicutes while reducing Bacteroidetes compared with the Control group (Fig. 4d). High-dose apiin treatment reversed these changes, markedly increasing Bacteroidetes and reducing Proteobacteria. At the level of genus, in the DSS group, the relative abundance of *Akkermansia* (belonging to Akkermansiaceae) and *Bacteroides* (belonging to Bacteroidaceae) was significantly reduced compared to the Control (Fig. 4e and Table S4). Notably, the apiin intervention significantly increased the relative abundance of *Akkermansia* and *Bacteroides* while suppressing *Escherichia* (belonging to Enterobacteriaceae) (Fig. 4f,g). Furthermore, LEfSe analysis confirmed that the enrichment of *Akkermansia* and *Bacteroides* was a defining feature of the gut microbiota following apiin treatment (Fig. 4h).

### Apiin altered microbial functional and metabolic profiles

**Fig. 5:**
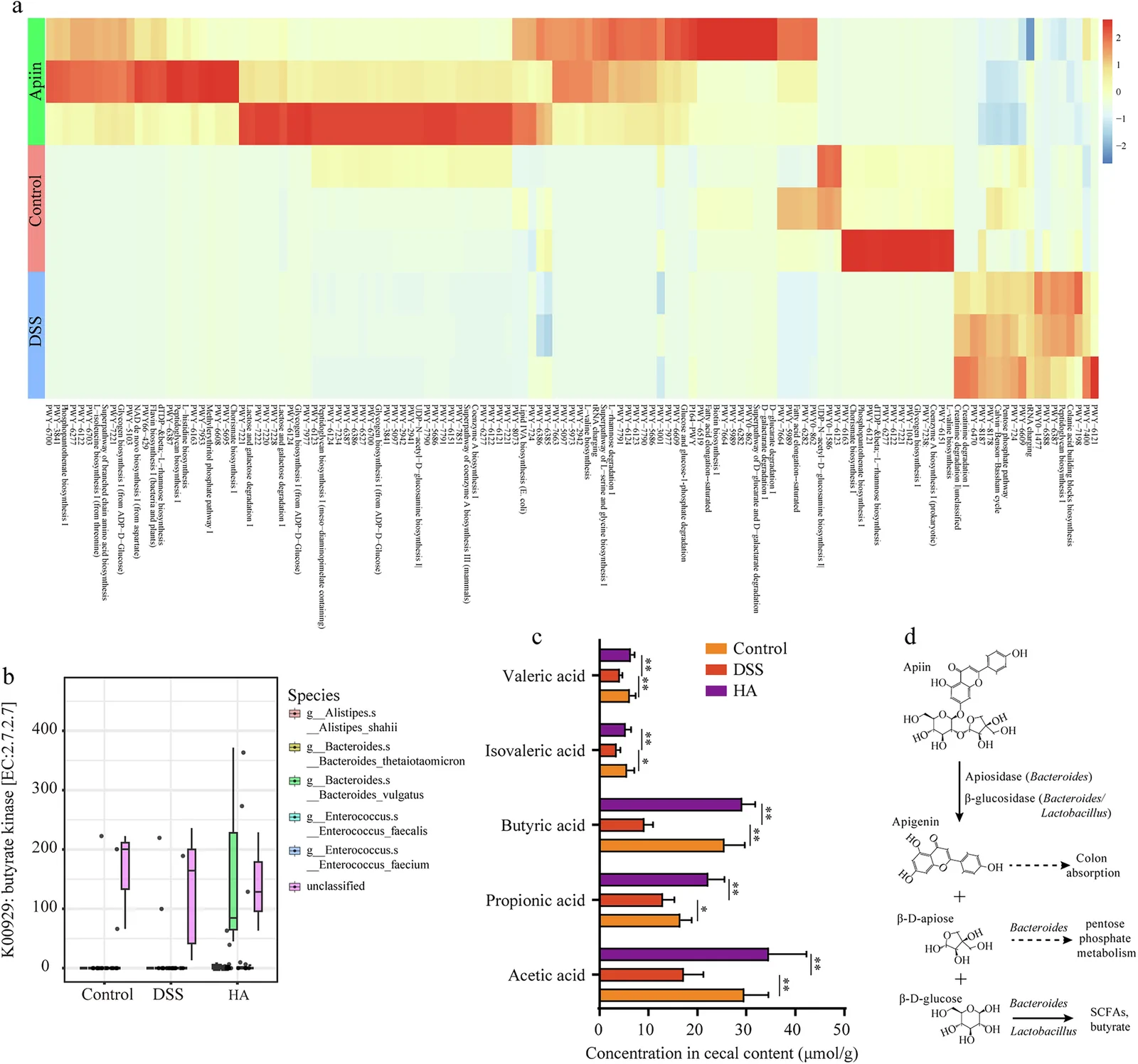
Apiin treatment modulates microbial functional pathways and short-chain fatty acid metabolism in DSS-induced colitis mice. **a** Relative abundance of MetaCyc pathways identified using the HUMAnN pipeline under default parameters. **b** Relative abundance of the butyrate kinase enzyme (K00929) in each groups. **c** Concentrations of major SCFAs in fecal samples. **d** Schematic metabolic diagram illustrating the proposed microbial biotransformation pathway of apiin in the gut microbiota. *p < 0.05, **p < 0.01; ns, not significant.

To investigate the interplay between apiin treatment and gut microbiota alterations, we first profiled gene families in all metagenomes using HUMAnN3 and aggregated their abundances according to KEGG Orthology (KO) annotations. Kruskal–Wallis analysis identified 459 enzymes that differed significantly between the DSS and HA groups (*p* < 0.05; Table S5). Functional attributions at the species level indicated that many of these enzymes were linked to carbohydrate and amino acid metabolism, with a notable enrichment in the apiin-treated group (Fig. 5a). Of particular interest, enzymes associated with butyrate production including acetyl-CoA C-acetyltransferase, L-lactate dehydrogenase, butyrate kinase, and acetate CoA/acetoacetate CoA-transferase, were markedly depleted in DSS-treated mice but significantly enriched following the apiin intervention (Fig. S1). Additionally, functional taxonomic profiling suggested that several enzymes enriched following apiin treatment were affiliated with *Bacteroides*. For example, butyrate kinase (K00929), encoded by the *buk* gene, exhibited increased abundance in the apiin-treated group (Fig. 5b). SCFA-targeted metabolomics further confirmed that the treatment of apiin significantly promoted the yield of butyrate (Fig. 5c). Collectively, these results demonstrated that apiin administration was associated with enhanced SCFA production in the intestine by enriching SCFA-producing genera and promoting the expression of butyrate-associated enzymes (Fig. 5d).

### Apiin significantly reduced the inflammatory response in colonic tissue of colitis mouse

**Fig. 6:**
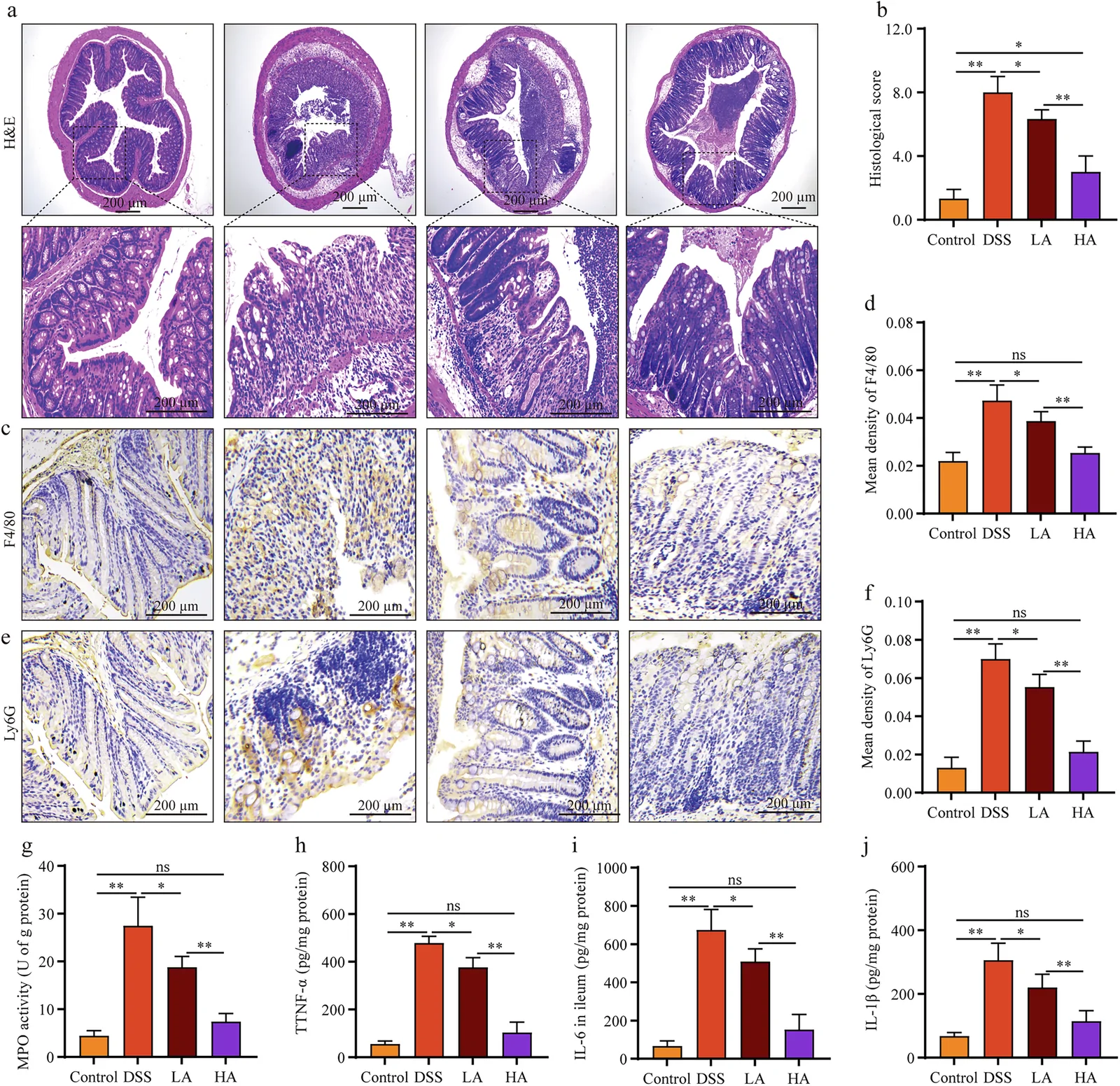
Apiin treatment mitigates intestinal inflammation in DSS-induced colitis mice. **a** Representative hematoxylin and eosin (H&E)–stained images of colonic tissues. **b** Histological scores of colon sections. **c**, **d** Immunohistochemical staining and quantitative analysis of F4/80 expression in colonic tissues. **e**, **f** Immunohistochemical staining and quantitative analysis of Ly6G expression in colonic tissues. **g** The MPO activity in colonic tissues. **h**–**j** Concentrations of TNF-α, IL-1β, and IL-6 in colonic tissues, respectively. *p < 0.05, **p < 0.01; ns, not significant.

DSS treatment often resulted in severe colonic injury^21^, which was confirmed by hematoxylin and eosin (H&E) staining in this study (Fig. 6a). In the representative sections of H&E staining, we observed notable epithelial disruption, inflammatory cell infiltration, and crypt loss in DSS group of mice, while the administration of high-dose apiin markedly alleviated these histological abnormalities (*p* < 0.01; Fig. 6a,b). We further evaluated the infiltration of macrophages (F4/80⁺ cells) and neutrophils (Ly6G⁺ cells) in colonic tissues using immunohistochemistry (IHC). DSS treatment significantly increased the infiltration of both macrophages and neutrophils, consistent with the histological injury observed on H&E staining (*p* < 0.01; Fig. 6a–f). Low-dose apiin treatment produced moderate improvements in colonic histology and slightly reduced immune cell infiltration. While the administration of high-dose apiin significantly prevented macrophage and neutrophil infiltration, which was accompanied by reduced histological injury and lower secretion of the pro-inflammatory cytokines TNF-α, IL-1β, and IL-6 in colonic tissue (*p* < 0.01; Fig. 6g–i). Consistent with these findings, mRNA expression levels of inflammatory cytokine genes *TNF-α*, *IL-1β*, and *IL-6* further confirmed that high-dose apiin suppressed the inflammatory response in DSS-induced colitis (*p* < 0.01; Fig. S2).

### Apiin markedly improved intestinal barrier function in colitis mice

**Fig. 7:**
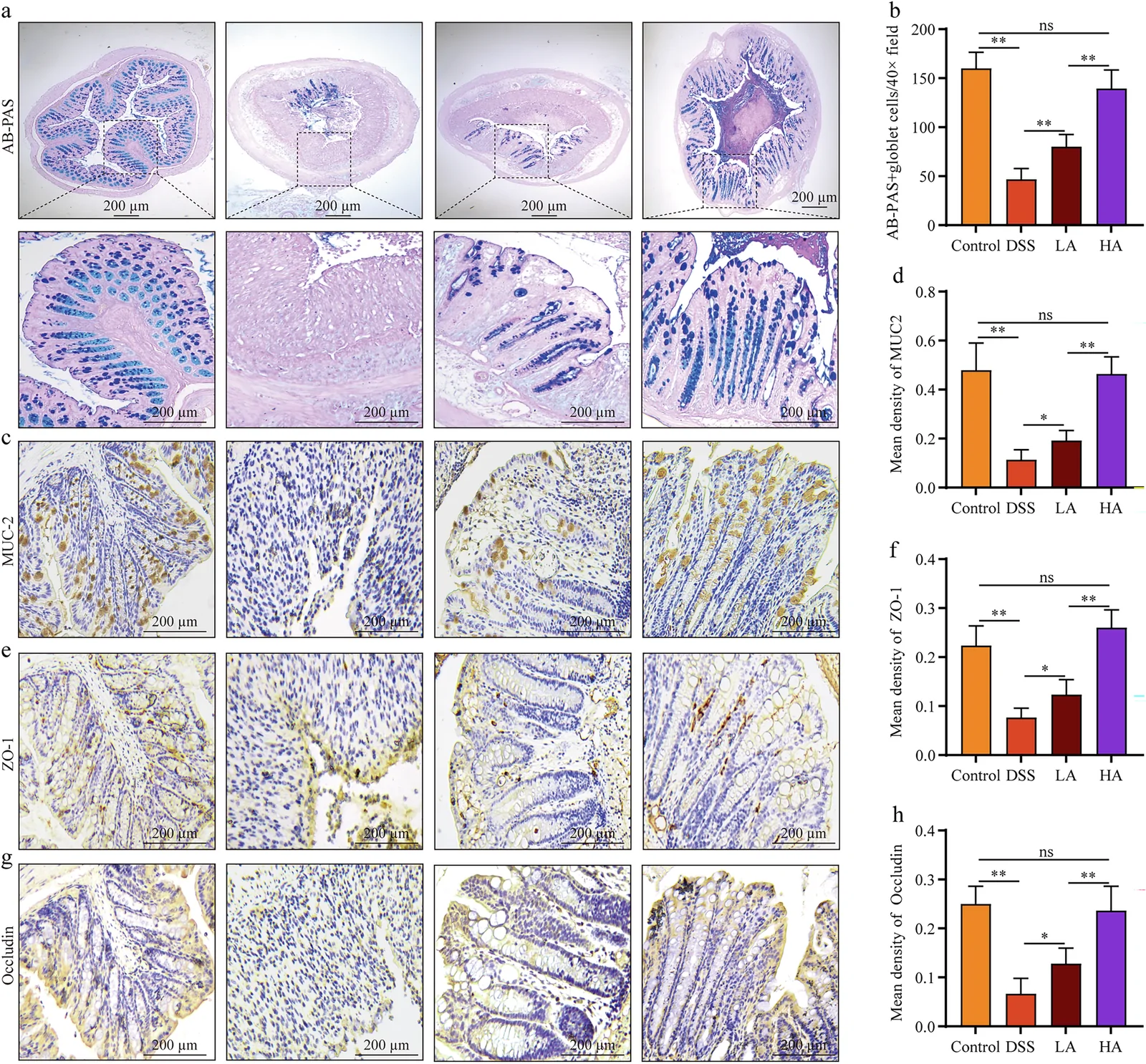
Apiin treatment enhances intestinal barrier integrity in DSS-induced colitis mice. **a** Representative images of alcian blue/periodic acid–Schiff (AB-PAS)–stained colonic sections. **b** Quantitative analysis of AB-PAS–positive goblet cells in colonic tissues. **c**, **d** Immunohistochemical staining and quantitative analysis of MUC2 expression in colonic tissues. **e**, **f** Immunohistochemical staining and quantitative analysis of ZO-1 expression in colonic tissues. **g**, **h** Immunohistochemical staining and quantitative analysis of occludin expression in colonic tissues. *p < 0.05, **p < 0.01; ns, not significant.

Impaired intestinal barrier integrity is a critical factor in the onset and progression of colitis^22^. Consistent with this, DSS treatment significantly increased serum LPS levels, reflecting compromised barrier function, whereas the administration of high-dose apiin effectively prevented this increase (*p* < 0.01; Fig. S3). Additionally, alcian blue/periodic acid–Schiff (AB–PAS) staining revealed a marked reduction in goblet cell numbers in DSS-treated mice compared with the control mice (*p* < 0.01; Fig. 7a,b). Apiin treatment, particularly at a high dose, significantly restored goblet cell abundance to levels equivalent to those observed in the control group. This effect was further supported by the increased expression of MUC2 protein in colonic tissues of the HA group (*p* < 0.01; Fig. 7c,d). We next evaluated tight junction proteins by IHC. DSS treatment markedly reduced the expression of ZO-1 and occludin, whereas the treatment of high-dose apiin significantly restored the expression of both proteins (*p* < 0.01; Fig. 7e–h). Consistently, mRNA expression of *ZO-1*, *occludin*, and *MUC2* further confirmed that high-dose apiin markedly improved intestinal barrier function in DSS-induced colitis (*p* < 0.01; Fig. S4).

### Apiin-associated butyrate enrichment likely participated in alleviating mouse colitis

**Fig. 8:**
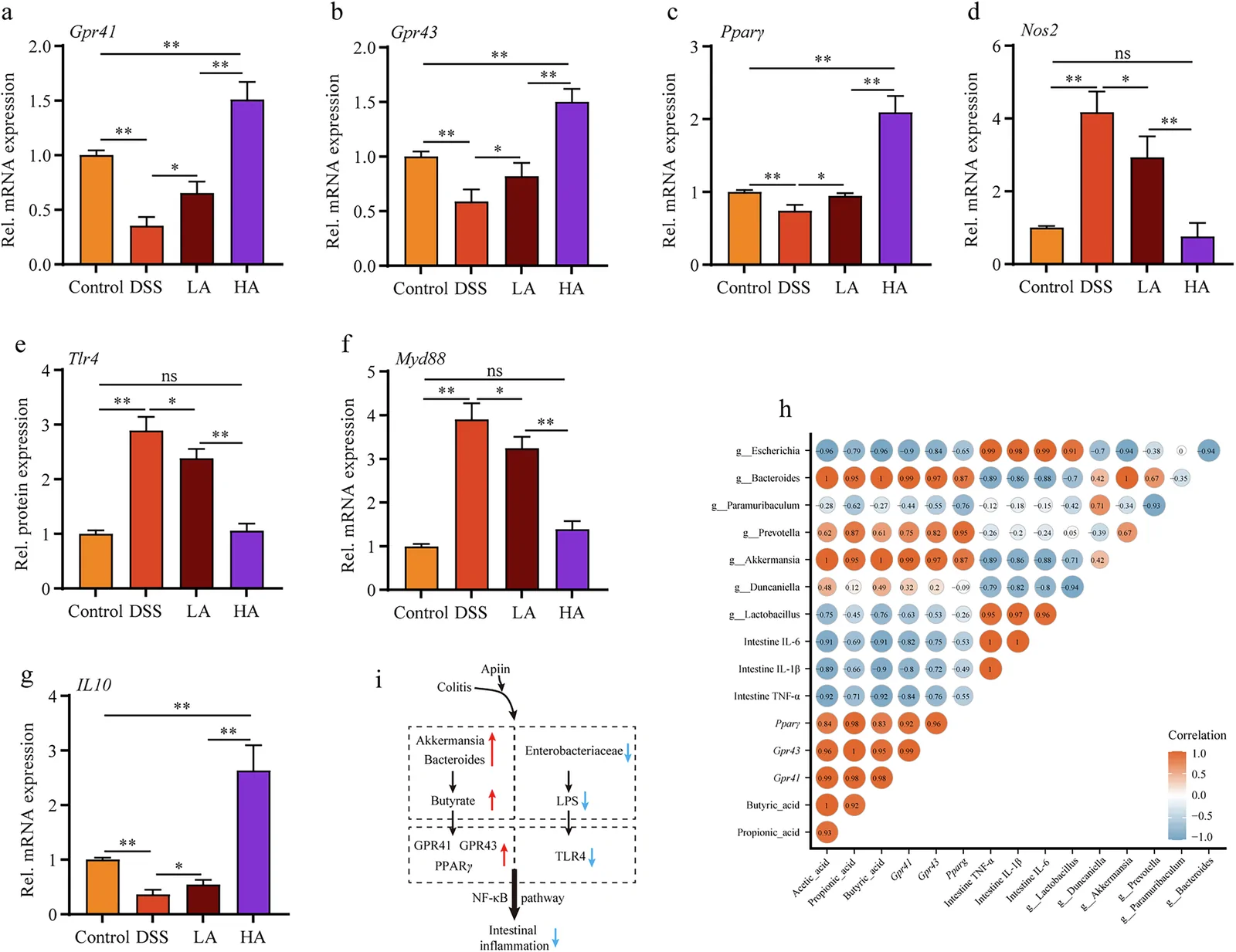
Apiin alleviates intestinal inflammation through butyrate-associated signaling pathways in DSS-induced colitis mice. Relative mRNA expression of butyrate receptor genes: **a** *Gpr41*, **b** *Gpr43*, **c** *Pparγ*, and **d** *Nos2*. Expression of genes involved in the TLR4 signaling pathway: **e** *Tlr4* and **f** *Myd88*. **g** Expression of anti-inflammatory cytokine gene *Il10*. **h** Heatmap showing correlations among representative gut microbiota, inflammatory cytokines, butyrate receptor expression, and short-chain fatty acid (SCFA) levels. **i** Proposed schematic model illustrating the mechanism by which apiin ameliorates DSS-induced colitis via butyrate enrichment and inflammation suppression. *p < 0.05, **p < 0.01; ns, not significant.

To explore whether the apiin-associated enrichment of butyrate drives its protective effects against colitis, we examined the expression of major butyrate-responsive GPRs, including *Gpr41* (encoding the metabolic regulator GPR41) and *Gpr43* (encoding the immunomodulatory receptor GPR43)^23^. Reverse transcription quantitative PCR (RT-qPCR) analysis showed that DSS treatment markedly downregulated *Gpr41* and *Gpr43* expression compared with controls (*p* < 0.01; Fig. 8a,b). In contrast, high-dose apiin treatment significantly upregulated both receptors, correlating positively with restored butyrate levels in cecal contents. We further assessed the expression of *Pparγ*, which encodes peroxisome proliferator-activated receptor gamma (PPARγ), another key receptor associated with butyrate in intestinal tissues^24^. DSS treatment reduced *Pparγ* expression, which was restored by the intervention of high-dose apiin to that of the control group (Fig. 8c,d). Phosphorylated p65 was also decreased in the colonic tissues of mice in the HA group (Fig. S5). Additionally, apiin treatment modulated *Nos2* expression and attenuated colonic inflammation.

Endotoxemia caused by LPS derived from intestinal microbiota is a major driver of colitis^25^. In the present study, DSS treatment significantly increased serum LPS levels and upregulated the expression of the key signaling mediators Tlr4 (encoding toll-like receptor 4, TLR4) and *Myd88* (encoding myeloid differentiation primary response 88, MYD88) (Fig. 8e,f). Notably, high-dose apiin markedly reduced serum LPS concentrations to the levels of the control group and suppressed the expression of its targeted genes *Tlr4* and *Myd88* in colonic tissues. Moreover, correlation analyses further confirmed that SCFAs, particularly butyrate, were associated with the relative abundances of *Akkermansia* and *Bacteroides* (*r* > 0.6, *p* < 0.05), as well as with increased expression of *Gpr41*, *Gpr43*, and *Pparγ*. Conversely, these parameters were negatively correlated with proinflammatory cytokines TNF-α, IL-1β, and IL-6 in colonic tissues (*r* < –0.6, *p* < 0.05; Fig. 8g,h).

### The gut microbiota was essential for apiin’s protective effects against colitis in mice

**Fig. 9:**
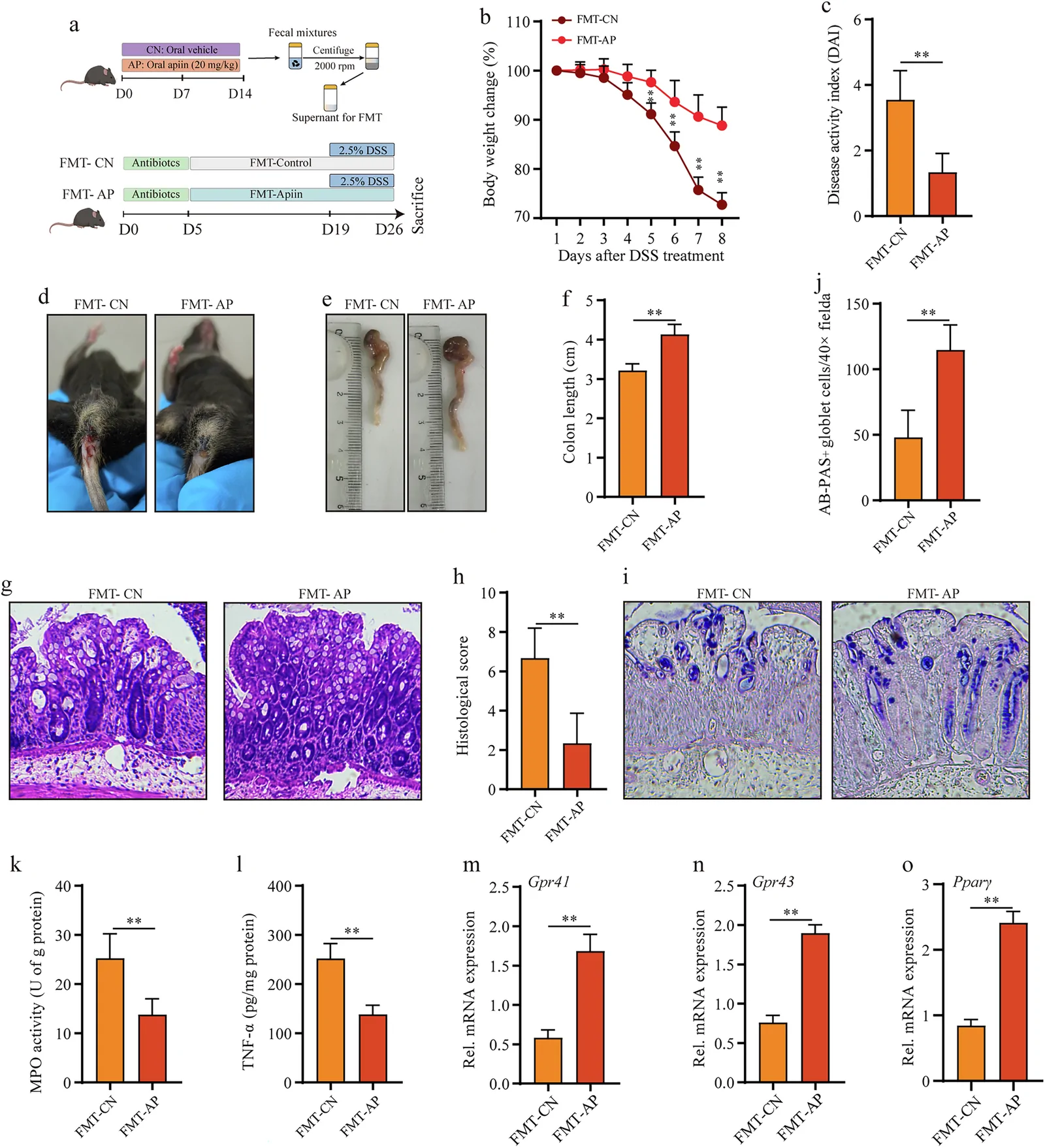
Fecal microbiota transplantation confirms the essential role of gut microbiota in apiin-mediated alleviation of DSS-induced colitis in mice. **a** Schematic illustration of the fecal microbiota transplantation (FMT) experimental design. **b** Body weight changes during DSS treatment. **c** Disease activity index (DAI) scores. **d** Representative images showing fecal occult blood and hematochezia. **e** Representative images of colon morphology. **f** Statistical analysis of colon length. **g** Representative hematoxylin and eosin (H&E)-stained images of colonic tissues. **h** Histological scores of colon sections. **i** Representative alcian blue/periodic acid–Schiff (AB-PAS)–stained images of colonic tissues. **j** Quantitative analysis of AB-PAS–positive goblet cells. **k** MPO activity in colonic tissues. **l** Concentrations of TNF-α in colonic tissues. Relative mRNA expression levels of butyrate receptor genes: **m** *Gpr41*, **n** *Gpr43*, and **o** *Pparγ* compared to the control mice. **p < 0.01.

To determine whether the gut microbiota mediated the anti-colitic effects of apiin, FMT experiments were conducted (Fig. 9a). Fecal contents from donor mice treated with high-dose apiin (FMT-AP) or control mice (FMT-CN) were transplanted into recipient mice with DSS-induced colitis. Mice receiving fecal microbiota from the apiin-treated donors (FMT-AP) exhibited a significant increase in body weight on day 5 after DSS treatment, compared with the FMT-CN group (Fig. 9b). In parallel, the DAI score of FMT-AP mice was markedly reduced (*p* < 0.01; Fig. 9c). Fecal observations further supported this finding that feces from the FMT-CN group often contained visible blood, whereas those from the FMT-AP group were well-formed and the perianal regions remained dry (Fig. 9d).

Histological analyses corroborated the physiological findings. Compared with FMT-CN mice, FMT-AP recipients exhibited significantly less colon shortening and improved tissue architecture, characterized by intact epithelial structures, reduced inflammatory infiltration, and preserved crypts (*p* < 0.01; Fig. 9e–h). In addition, transplantation of apiin-associated microbiota enhanced mucosal integrity, increased villus density, and promoted mucin secretion in colonic tissues, as evidenced by AB-PAS staining (*p* < 0.01; Fig. 9i,j). Next, we assessed inflammatory responses in the colon. FMT-AP mice showed markedly decreased levels of myeloperoxidase (MPO) and TNF-α, compared with FMT-CN mice (*p* < 0.05; Fig. 9k,l). Furthermore, the expression of butyrate-responsive receptor genes–including *Gpr41*, *Gpr43*, and *Pparγ*–was significantly upregulated in the FMT-AP group (*p* < 0.01; Fig. 9m-o), positively correlating with elevated fecal butyrate concentrations (Fig. S6). Collectively, these results of fecal microbiota transplantation (FMT) demonstrated that the gut microbiota played a pivotal role in mediating the therapeutic effects of apiin against colitis.

## Discussion

Inflammatory bowel disease arises from a dynamic interplay between host immunity and the gut microbiota, in which microbial dysbiosis and impaired metabolic outputs are central drivers of mucosal inflammation^26–28^. In the present study, we identify apiin as a microbiota-responsive dietary flavone glycoside that confers protection against DSS-induced colitis through coordinated modulation of microbial composition, metabolic function, and host signaling pathways. By integrating chemical characterization, gastrointestinal tracking, and multi-omics analyses, we provide a mechanistic framework linking apiin biotransformation to microbiota-dependent anti-inflammatory effects.

A key finding of this study is that apiin functions as a colon-delivered pro-flavonoid, remaining largely intact in the upper gastrointestinal tract and undergoing selective deglycosylation in the distal intestine. This spatially restricted metabolism highlights the importance of gut microbiota in unlocking the bioactivity of glycosylated flavonoids. Notably, unlike apigetrin (apigenin-7-O-glucoside), which contains a single glucose moiety and can be more readily hydrolyzed and absorbed in the ileum region, apiin possesses a more complex apiose-glucose disaccharide that confers increased resistance to host digestion and promotes delayed microbial conversion^29,30^. The delayed appearance of apigenin in the ileum, cecum, and colon, together with its accumulation in colonic tissues, supports a model in which microbial enzymes mediate apiin hydrolysis, thereby generating bioactive metabolites at sites of inflammation. This feature distinguishes apiin from both readily absorbable aglycones and simpler glycosides such as apigetrin, underscoring its potential as a microbiota-targeted dietary modulator of colonic health. Furthermore, the effective dose used in the present study (20 mg/kg in mice) corresponds to an estimated human equivalent dose of approximately 1.62 mg/kg (~113 mg/day for a 70-kg adult), suggesting that the observed effects may be nutritionally and translationally relevant. Given that apiin is naturally abundant in commonly consumed plant-based foods such as parsley and celery, its biological activity may potentially be achieved through dietary intake or nutraceutical supplementation strategies targeting intestinal health.

Beyond biotransformation, apiin markedly reshaped the gut microbial ecosystem disrupted by DSS. Specifically, apiin restored microbial diversity and promoted the enrichment of *Akkermansia* and *Bacteroides*, taxa widely associated with mucosal homeostasis and metabolic flexibility^31,32^. The expansion of *Akkermansia*, particularly *Akkermansia muciniphila*, is notable given its established roles in mucin turnover, epithelial repair, and immune modulation^33^. Concurrently, *Bacteroides* species, as key degraders of complex carbohydrates, likely contributed to enhanced fermentation capacity and short-chain fatty acid (SCFA) production^34^. These findings support the concept that apiin acts as a microbiota-accessible substrate, selectively promoting beneficial microbial consortia and restoring ecological balance.

Functionally, our multi-omics analyses revealed a pronounced shift toward SCFA-centered metabolism, with butyrate emerging as a critical effector molecule. Apiin treatment enriched microbial genes encoding key enzymes in butyrate biosynthesis pathways, and this was corroborated by increased luminal butyrate levels. Given the central role of butyrate in maintaining intestinal homeostasis, these results suggest that apiin exerts a prebiotic-like effect, enhancing microbial metabolic outputs rather than acting solely through direct host-target interactions.

Mechanistically, the elevation of butyrate was tightly linked to the activation of host signaling pathways involved in immune regulation and barrier function^35^. Apiin restored the expression of butyrate-responsive receptors GPR41 and GPR43, as well as the nuclear receptor PPARγ, all of which were suppressed under DSS-induced inflammatory conditions. Activation of these pathways is known to inhibit NF-κB signaling, thereby reducing proinflammatory cytokine production and promoting epithelial integrity^36–38^. Consistent with this mechanism, apiin treatment was associated with reduced expression of TNF-α, IL-1β, and IL-6, and reduced immune cell infiltration. In parallel, apiin reinforced barrier function, as evidenced by increased goblet cell abundance, enhanced MUC2 secretion, and restoration of tight junction proteins ZO-1 and occludin, alongside reduced systemic LPS levels. Together, these findings support a microbiota–butyrate–host signaling axis as a central mechanism underlying the anti-colitic effects of apiin.

Importantly, fecal microbiota transplantation (FMT) experiments established a causal role for the microbiota in mediating these protective effects. Transfer of microbiota from apiin-treated donors recapitulated improvements in disease severity, mucosal integrity, and inflammatory markers in recipient mice. This provides strong evidence that apiin does not act solely as a direct anti-inflammatory compound but rather exerts its effects through microbiota-driven metabolic reprogramming. Such findings align with an emerging paradigm in food science, wherein the health benefits of dietary phytochemicals are increasingly attributed to their interactions with and transformation by the gut microbiome.

Despite these advances, several limitations should be acknowledged. The respective contributions of apiin-derived apigenin and microbiota-associated butyrate enrichment were not independently dissected, and direct butyrate dependency was not experimentally validated. Although butyrate-associated pathways were partially attributed to *Bacteroides* species, their precise role in intestinal butyrate production remains to be established. In addition, microbial compositional changes in FMT recipients and the specific microbial enzymes responsible for apiin deglycosylation were not directly characterized. Mechanistic interpretations were primarily based on transcriptional and expression-level analyses, and therefore require further functional validation of GPR41/GPR43–PPARγ and NF-κB signaling. Moreover, microbiome and metabolomic analyses were limited to the high-dose apiin group, leaving potential dose-dependent effects unresolved, while SCFA measurements in cecal contents may not fully reflect the metabolic environment of the distal colon. Finally, because the acute DSS model predominantly reflects epithelial injury and innate immune activation, further studies in chronic and immune-mediated colitis models will be necessary to establish broader relevance to human IBD, and potential sex-dependent responses to apiin will be investigated in future study.

**Fig. 10:**
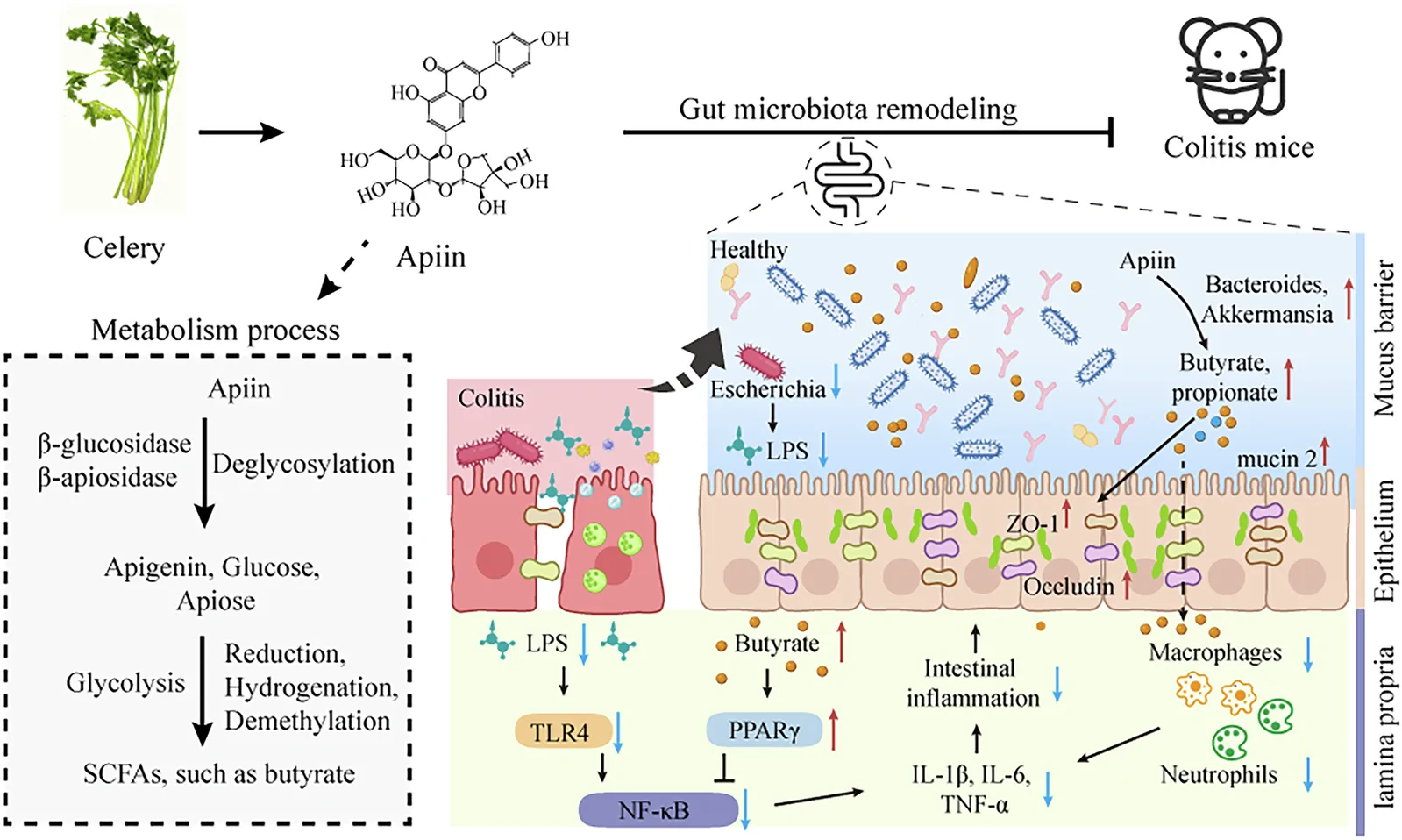
Microbiota-dependent biotransformation of apiin alleviates colitis. Orally administered apiin reaches the distal intestine and undergoes microbiota-dependent deglycosylation to apigenin. Apiin reshapes gut microbiota, enriching *Akkermansia* and *Bacteroides* and enhancing butyrate production. Increased butyrate levels were associated with activation of GPR41/43 and PPARγ and reduced inflammation.

In conclusion, this study demonstrates that apiin acts as a microbiota-dependent pro-flavonoid that alleviates colitis in association with coordinated modulation of microbial composition and metabolic function (Fig. 10). The microbial biotransformation of apiin into apigenin was accompanied by enhanced butyrate production and increased expression of GPR41/GPR43 and PPARγ genes, which were associated with reduced inflammation and improved intestinal barrier integrity. Collectively, these findings provide insight into how dietary glycosides interact with the gut microbiota to influence host physiology and highlight apiin as a promising candidate for the development of microbiome-targeted functional foods for intestinal inflammatory disorders.

## Methods

### Animal model of colitis and experimental design

Male C57BL/6J mice (5–6 weeks old, 18–20 g) were obtained from Charles River Laboratories (Beijing, China). Mice were acclimated for one week in a specific pathogen-free (SPF) facility under controlled conditions (temperature, 24 ± 1 °C; humidity, 55 ± 5%; 12 h light/dark cycle) with ad libitum access to food and water. All animal procedures were approved by the Institutional Animal Care and Use Committee of Nankai University and conducted in accordance with the National Guidelines for the Care and Use of Laboratory Animals (Approval No: 2022-SYDELL-000530). Following acclimatization (3 days), mice were randomly assigned to experimental groups using a random-number-based allocation procedure (*n* = 8 per group): (1) Control**:** normal drinking water; (2) DSS: 2.5% (w/v) DSS in drinking water; (3) LA: DSS + low-dose apiin (2 mg/kg/day); (4) HA: DSS + high-dose apiin (20 mg/kg/day). DSS was administered from days 1–7 to induce acute colitis, followed by normal water from days 8–10. Apiin (Aladdin, ≥97% purity, Shanghai, China) was delivered daily via oral gavage throughout the DSS exposure period (days 1–7), representing a concurrent intervention during colitis induction. And the selected apiin doses (2 and 20 mg/kg/day) were based on preliminary dose-screening experiments and previous studies of flavonoids and related dietary phytochemicals in murine models. The Control and DSS model groups received equal volumes of vehicle (0.5% CMC-Na). On day 11, mice were anesthetized with isoflurane (2%) and euthanized with cervical dislocation. Colon length was measured, and serum, colon tissues, and cecal contents were collected for subsequent analyses. Unless otherwise indicated, analyses were performed using biological replicates from all mice in each group; and no animals or samples were excluded from analysis.

### Fecal microbiota transplantation (FMT)

FMT was performed following modified published protocols^39^. To deplete native gut microbiota, recipient mice received an antibiotic cocktail (metronidazole, penicillin, streptomycin, neomycin, each 0.5 g/L; vancomycin, 0.25 g/L) in drinking water for 5 days prior to the FMT procedure. Microbial clearance was verified by plating fecal suspensions on nutrient agar under anaerobic conditions. Donor feces were obtained from apiin–treated (20 mg/kg/day) and vehicle-treated mice after 14 days of intervention. Fresh feces (0.1 g) were suspended in 1 mL sterile phosphate-buffered saline (PBS), vortexed, and centrifuged at 500 × *g* for 5 min. Recipient mice were given 200 μL of the fecal supernatant daily through oral gavage for 21 days, and all mice underwent 2.5% DSS treatment from day 19 to day 26. After treatment, samples of colon tissue, serum, and cecal content were collected analyzed as described above.

### Disease activity index (DAI)

DAI was determined daily based on percentage body weight loss, stool consistency, and fecal occult blood, as previously described^40^. Each parameter was scored from 0 to 4, and the total DAI score represented the mean of all criteria (Table S1). All DAI scoring, histological assessments, and immunohistochemical quantifications were performed by investigators blinded to group allocation.

### Histological and immunohistochemical analyses

Colonic tissues were fixed in 4% paraformaldehyde, dehydrated, and embedded in paraffin. Sections (5 μm) were stained with H&E for histopathology or AB–PAS for goblet cell assessment. Histological scoring was performed by blinded observers based on epithelial damage, inflammatory infiltration, and ulceration, using established criteria (Table S2).

For IHC, deparaffinized sections were subjected to antigen retrieval, endogenous peroxidase quenching (3% H₂O₂), and blocking with 5% goat serum. The slides were then incubated overnight at 4 °C with primary antibodies against ZO-1, Occludin, MUC-2, p-p65, F4/80, or Ly6G (1:200, Affinity), followed by HRP-conjugated secondary antibodies and DAB chromogenic detection. Images were acquired using an Olympus light microscope.

### Enzyme-linked immunosorbent assay (ELISA)

Colonic tissues were homogenized in nine volumes of ice-cold PBS and centrifuged (10,000 × *g*, 5 min, 4 °C). Serum was obtained by centrifugation of whole blood (1500 × *g*, 15 min, 4 °C). Supernatants were used for ELISA quantification of TNF-α, IL-1β, IL-6, and MPO according to manufacturer protocols (Solarbio, Beijing, China). Absorbance was measured using a microplate reader (SpectraMax ID3, Molecular Devices), and concentrations were calculated from standard curves. Tissue cytokine levels were normalized to tissue weight (pg/mg protein), whereas serum cytokine concentrations were expressed as pg/mL.

### RT-qPCR experiments

Total RNA was extracted from colonic tissues using TRIzol reagent (Invitrogen, Cat. No. 15596018CN). cDNA synthesis and amplification were performed with PrimeScript RT Reagent Kit and SYBR Green PCR Master Mix (Takara, Japan). Reactions were carried out in a 20 μL volume on a QuantStudio 3 system (Applied Biosystems). Relative mRNA expression was normalized to 18S gene using the 2^−ΔΔCt^ method^41^. Primer sequences are listed in Table S3.

### Gut microbiota profiling

Metagenomic sequencing was performed using cecal contents from 5 mice per group. Fecal DNA was extracted using the DNA Stool Kit (Solarbio, Beijing, China). DNA concentration and purity were verified using a NanoPhotometer N50 (Implen, Germany) and 1% agarose gel electrophoresis. Libraries were prepared with the NEBNext Ultra DNA Library Prep Kit (New England Biolabs) and sequenced on an Illumina NovaSeq 6000 platform (6 G, PE150). To obtain clean data, low-quality reads were trimmed using Trimmomatic (v0.40)^42^, and host sequences were removed by alignment to the mouse genome (GRCm39) using Bowtie2 (v2.3.5.1)^43^; an average of 23.6 million paired-end reads per sample was generated. Taxonomic profiling was performed with MetaPhlAn (v4.2.2)^44^, and functional annotation was conducted using HUMAnN (v3.0)^45^. Alpha diversity indices were calculated using the “calculate_diversity.R” package, and beta diversity was analyzed via Bray–Curtis distances and principal coordinate analysis (PCoA). Visualization and statistical analyses were performed using “vegan” and “ggplot2” packages in R. For omics analyses, *p* values were adjusted for multiple comparisons using the Benjamini–Hochberg false discovery rate (FDR) correction, and adjusted *p* < 0.05 was considered statistically significant where applicable.

### Targeted metabolomics of SCFAs

Approximately 100 mg of cecal content was homogenized with 80% methanol, vortexed, and centrifuged (10,000 × *g*, 20 min, 4 °C). The supernatant was derivatized using 3-nitrophenylhydrazine (3-NPH) and EDC reagents, followed by LC–MS/MS analysis on a Thermo Fisher Q Exactive mass spectrometer (Waltham, Massachusetts, USA) equipped with a C18 column (250 mm, 4.6 mm; USA). Calibration curves for acetate, propionate, and butyrate were used for quantification, with QC samples ensuring analytical reproducibility (RSD < 15%).

### Statistical analysis

Data are presented as mean ± standard deviation (SD). Normality was assessed prior to statistical comparisons. Statistical analyses were performed using GraphPad Prism 9.0. Differences among multiple groups were assessed by one-way ANOVA followed by Tukey’s post hoc test for normally distributed data, while nonparametric tests were applied when appropriate. Correlation analyses were conducted using Pearson’s correlation coefficient. Values of *p* < 0.05 were considered statistically significant.

## Supplementary information

Supplementary information

## Data Availability

The datasets generated and/or analyzed during the current study are not publicly available because they are part of ongoing investigations and additional analyses, but are available from the corresponding author on reasonable request.
