# Supplementary material for: Microbiota-dependent biotransformation of apiin drives butyrate-associated protection against colitis
Source: NPJ Sci Food. 2026 Jul 14;10:348. doi: 10.1038/s41538-026-00992-4 (PMC13740267; doi:10.1038/s41538-026-00992-4)
Supplement: Supplementary file 1 [file 41538_2026_992_MOESM1_ESM.docx]

**Microbiota-dependent biotransformation of apiin drives butyrate-mediated protection against colitis**

Zhenning Guo^a#^, Meiqi Zhao^b#^, Yuwei Wu^a^, Peng Zhang^a^*, Shiming Li^c^*

^a^Tianjin Key Laboratory of Life and Health Detection, Life and Health Intelligent Research Institute, Tianjin University of Technology, Tianjin 300384, China;

^b^Department of Cardiology, Department of gastroenterology &hepatology, Tianjin First Center Hospital, Tianjin 300192, China;

^c^Department of Food Science, Rutgers University, New Brunswick, New Jersey 08901, United States; College of Biology and Agricultural Resources, Huanggang Normal University, Huanggang, Hubei 438000, China.

^#^These authors contribute equally to the work.

*Address corresponding to Peng Zhang, Email: zpeng@email.tjut.edu.cn, Tel.: +86-022-60216079; Shiming Li, Email: shiming@rutgers.edu.

The supplementary information includes three appendices. The first appendix contains information about additional methods and protocols. The second appendix provides details on additional figures. The third appendix describes supplementary text for experiments.

I. **Supplementary Figures**
II. **Supplementary Tables**
III. **Supplementary Texts**

## I. Supplementary Figures

Figure S1.The heatmap revealed the abundance of these genes associated with butyrate production in fecal samples accoss different groups.

Figure S2. Relative mRNA expression levels of proinflammatory cytokine genes, including *TNF-α*, *IL-1β*, and *IL-6*. **p* < 0.05, ***p* < 0.01; ns, not significant.

Figure S3. The serum concentration of LPS across different groups. **p* < 0.05, ***p* < 0.01; ns, not significant.

Figure S4. Relative mRNA expression levels of intestinal barrier genes, including *ZO-1*, *occludin*, and *MUC2*. **p* < 0.05, ***p* < 0.01; ns, not significant.

Figure S5. Immunohistochemical staining of MUC2 expression in colonic tissues accoss different groups.

Figure S6. Concentrations of major short-chain fatty acids (SCFAs) in fecal samples.

**II. Supplemental Tables**

Table S1. The relative abundance of top 200 genus identified in fecal metagenomes.

|  | Control | | DSS | | HA | |
| --- | --- | --- | --- | --- | --- | --- |
| clade_name | mean | sd | mean | sd | mean | sd |
| g__Lactobacillus | 17.77414 | 16.73832 | 10.91806 | 2.982759 | 11.71753 | 7.161342 |
| g__Duncaniella | 13.94855 | 5.583071 | 5.15526 | 0.832341 | 7.050083 | 5.252398 |
| g__Akkermansia | 3.1681 | 2.31662 | 4.128147 | 2.257142 | 8.736963 | 1.549046 |
| g__Muribaculaceae_unclassified | 4.538073 | 1.542604 | 1.778597 | 1.572004 | 6.500393 | 3.31899 |
| g__GGB14014 | 2.41607 | 1.387935 | 3.951807 | 0.637027 | 6.28285 | 1.905241 |
| g__Prevotella | 2.59888 | 1.807248 | 5.965517 | 4.128072 | 2.151867 | 2.606144 |
| g__Muribaculum | 6.17985 | 2.014296 | 1.054733 | 0.831596 | 2.624383 | 0.594148 |
| g__Ligilactobacillus | 1.78211 | 1.735403 | 0.384157 | 0.044937 | 6.940687 | 5.372596 |
| g__Paramuribaculum | 3.183947 | 2.197738 | 3.86942 | 0.886529 | 1.692113 | 1.799362 |
| g__GGB24132 | 2.466617 | 2.22257 | 1.4209 | 0.569639 | 4.387157 | 1.541216 |
| g__Bacteroidales_unclassified | 1.525997 | 2.211863 | 6.19552 | 4.154536 | 0.104457 | 0.166276 |
| g__Limosilactobacillus | 2.843907 | 2.335605 | 0.453597 | 0.443856 | 4.444617 | 4.123486 |
| g__Bacteroides | 3.206643 | 2.458367 | 2.406623 | 0.595024 | 1.598197 | 1.481767 |
| g__GGB27890 | 4.935753 | 6.767711 | 1.911917 | 1.081611 | 0.339407 | 0.113708 |
| g__GGB31822 | 2.713727 | 1.823867 | 0.742827 | 0.504561 | 3.457153 | 5.493099 |
| g__Bifidobacterium | 0.768483 | 0.968315 | 5.37506 | 2.621994 | 0.001047 | 0.001813 |
| g__GGB27885 | 0.04167 | 0.06973 | 5.77865 | 1.625687 | 0.113797 | 0.059009 |
| g__Rikenellaceae_unclassified | 1.509643 | 2.614779 | 3.285007 | 1.307731 | 0 | 0 |
| g__GGB31437 | 1.537533 | 1.035941 | 2.05192 | 0.919642 | 0.98534 | 0.171427 |
| g__GGB27929 | 1.467517 | 0.281281 | 0.765917 | 0.49288 | 1.739173 | 1.751839 |
| g__GGB27861 | 0.176097 | 0.253779 | 3.23392 | 2.010372 | 0.550237 | 0.948296 |
| g__GGB24146 | 0.005677 | 0.009832 | 0.007357 | 0.006488 | 3.89284 | 4.227645 |
| g__GGB24149 | 0 | 0 | 1.753943 | 0.466199 | 1.909323 | 3.295214 |
| g__GGB46152 | 0.902197 | 0.639098 | 1.968303 | 0.976154 | 0.689957 | 0.401868 |
| g__GGB14001 | 1.355503 | 2.156023 | 0.14938 | 0.080724 | 1.993497 | 1.889843 |
| g__GGB31701 | 1.61281 | 1.438174 | 0.43003 | 0.20504 | 1.367593 | 0.713371 |
| g__GGB28930 | 3.0542 | 4.96643 | 0 | 0 | 0 | 0 |
| g__GGB27872 | 0.338473 | 0.366204 | 2.59088 | 1.156183 | 0.033487 | 0.048376 |
| g__GGB33839 | 2.362337 | 1.136011 | 0.170397 | 0.123147 | 0.063243 | 0.085121 |
| g__Bacteria_unclassified | 0.847967 | 0.351036 | 0.479077 | 0.083081 | 0.829613 | 0.686671 |
| g__GGB27866 | 0.54439 | 0.088698 | 0.77583 | 0.297337 | 0.660967 | 0.501235 |
| g__GGB27860 | 0.621393 | 0.115999 | 0.883713 | 0.358214 | 0.457387 | 0.071382 |
| g__GGB27876 | 0.735823 | 0.245302 | 1.164417 | 1.003367 | 0 | 0 |
| g__Bacteroidaceae_unclassified | 0.62351 | 0.308154 | 0.593773 | 0.284932 | 0.491817 | 0.429193 |
| g__GGB31762 | 0.097517 | 0.067589 | 0.06383 | 0.067322 | 1.432883 | 2.088192 |
| g__GGB27776 | 0.44563 | 0.268809 | 1.12119 | 0.668714 | 0 | 0 |
| g__GGB25041 | 0 | 0 | 0 | 0 | 1.281767 | 1.90638 |
| g__GGB27782 | 0.56309 | 0.37049 | 0.501733 | 0.150292 | 0.216317 | 0.135499 |
| g__Alistipes | 0.00617 | 0.005811 | 1.021493 | 1.098741 | 0.084993 | 0.110918 |
| g__GGB27873 | 0.000977 | 0.001692 | 1.066933 | 1.417303 | 0.010653 | 0.010595 |
| g__Lachnospiraceae_unclassified | 0.102177 | 0.176975 | 0.293953 | 0.338341 | 0.62203 | 0.997435 |
| g__GGB47127 | 0 | 0 | 0.886543 | 0.686891 | 0.09095 | 0.080349 |
| g__GGB28895 | 0 | 0 | 0 | 0 | 0.96335 | 1.288301 |
| g__Adlercreutzia | 0.033267 | 0.016252 | 0.77236 | 1.183453 | 0.06673 | 0.037486 |
| g__GGB31439 | 0.020813 | 0.009559 | 0.744807 | 1.213108 | 0.018177 | 0.015627 |
| g__GGB27480 | 0.307317 | 0.125034 | 0.2811 | 0.181843 | 0.186317 | 0.0527 |
| g__Clostridiaceae_unclassified | 0.031753 | 0.054998 | 0.695623 | 1.067889 | 0.006417 | 0.011114 |
| g__GGB30473 | 0.221937 | 0.38118 | 0.4321 | 0.344176 | 0.06586 | 0.079527 |
| g__GGB30454 | 0.001787 | 0.003095 | 0.70205 | 0.759216 | 0.004747 | 0.008221 |
| g__GGB27778 | 0 | 0 | 0 | 0 | 0.706217 | 0.716972 |
| g__GGB27893 | 0.001357 | 0.001175 | 0.478113 | 0.401102 | 0.203917 | 0.178518 |
| g__GGB27481 | 0.16528 | 0.286273 | 0.3339 | 0.288279 | 0.163993 | 0.215042 |
| g__GGB25052 | 0.03966 | 0.045341 | 0.519757 | 0.633937 | 0.08008 | 0.111775 |
| g__GGB30620 | 0.025087 | 0.043451 | 0.487877 | 0.251174 | 0.058923 | 0.102058 |
| g__Parasutterella | 0.159333 | 0.139072 | 0.04155 | 0.064298 | 0.35626 | 0.41596 |
| g__GGB30470 | 0 | 0 | 0.498243 | 0.850699 | 0.03513 | 0.009017 |
| g__Mucispirillum | 0.09251 | 0.03572 | 0.31792 | 0.525168 | 0.121513 | 0.155371 |
| g__GGB28950 | 0.009887 | 0.017124 | 0 | 0 | 0.510527 | 0.882587 |
| g__GGB24102 | 0.13881 | 0.195694 | 0.113057 | 0.008597 | 0.253847 | 0.218385 |
| g__Clostridiales_unclassified | 0.42815 | 0.741578 | 0.063183 | 0.109437 | 0 | 0 |
| g__GGB30048 | 0.11476 | 0.111991 | 0.18126 | 0.096453 | 0.19169 | 0.282759 |
| g__GGB27792 | 0 | 0 | 0.47888 | 0.670573 | 0 | 0 |
| g__GGB29622 | 0.057837 | 0.065854 | 0.209803 | 0.142383 | 0.20145 | 0.343256 |
| g__GGB28994 | 0.064323 | 0.056431 | 0.359707 | 0.593052 | 0.0142 | 0.019104 |
| g__GGB28904 | 0.19904 | 0.094657 | 0.13951 | 0.064465 | 0.075587 | 0.058386 |
| g__GGB29685 | 0 | 0 | 0.336013 | 0.579681 | 0.058913 | 0.102041 |
| g__Enterorhabdus | 0 | 0 | 0.38939 | 0.673621 | 0 | 0 |
| g__GGB28901 | 0 | 0 | 0.375273 | 0.649542 | 0.012793 | 0.019618 |
| g__GGB28851 | 0.155663 | 0.269617 | 0.035073 | 0.023663 | 0.195217 | 0.329408 |
| g__GGB28955 | 0 | 0 | 0.21502 | 0.08013 | 0.159057 | 0.156539 |
| g__GGB28836 | 0.0696 | 0.07374 | 0.105273 | 0.037626 | 0.19225 | 0.091687 |
| g__GGB28927 | 0 | 0 | 0.36152 | 0.354975 | 0 | 0 |
| g__GGB28990 | 0 | 0 | 0 | 0 | 0.355743 | 0.242467 |
| g__GGB28759 | 0 | 0 | 0 | 0 | 0.32251 | 0.314194 |
| g__GGB28317 | 0.084117 | 0.142665 | 0.202137 | 0.098286 | 0.036033 | 0.058744 |
| g__Phocaeicola | 0.04598 | 0.047598 | 0.266377 | 0.206882 | 0 | 0 |
| g__GGB28765 | 0.243653 | 0.387878 | 0.006837 | 0.008342 | 0.060447 | 0.071401 |
| g__GGB31109 | 0.30884 | 0.416068 | 0 | 0 | 0 | 0 |
| g__GGB30145 | 0.042753 | 0.043763 | 0.257177 | 0.35723 | 0.008073 | 0.010415 |
| g__Atopobiaceae_unclassified | 0.072727 | 0.052641 | 0.031083 | 0.031185 | 0.195203 | 0.307854 |
| g__GGB28934 | 0.11667 | 0.1012 | 0.027473 | 0.006411 | 0.140657 | 0.210868 |
| g__GGB31179 | 0.05132 | 0.06546 | 0 | 0 | 0.217557 | 0.376819 |
| g__Emergencia | 0.25724 | 0.199577 | 0 | 0 | 0.001613 | 0.002132 |
| g__GGB29549 | 0.01782 | 0.027764 | 0.0328 | 0.030204 | 0.20041 | 0.271443 |
| g__GGB30460 | 0.096083 | 0.091268 | 0 | 0 | 0.150727 | 0.236238 |
| g__GGB30311 | 0.208097 | 0.102031 | 0.013503 | 0.012925 | 0.025103 | 0.031355 |
| g__GGB28769 | 0.07239 | 0.086195 | 0.125107 | 0.073625 | 0.045427 | 0.073437 |
| g__GGB28383 | 0.01001 | 0.009459 | 0.038793 | 0.045803 | 0.184533 | 0.068502 |
| g__GGB29531 | 0.087657 | 0.122926 | 0 | 0 | 0.14059 | 0.243509 |
| g__Eggerthellaceae_unclassified | 0.16363 | 0.183398 | 0.005793 | 0.000511 | 0.050493 | 0.038906 |
| g__GGB28946 | 0 | 0 | 0 | 0 | 0.218337 | 0.37817 |
| g__GGB28980 | 0.07696 | 0.087784 | 0.11174 | 0.169269 | 0.027087 | 0.036835 |
| g__GGB28949 | 0.060237 | 0.056406 | 0.034607 | 0.016355 | 0.108603 | 0.110834 |
| g__GGB30667 | 0.050493 | 0.087457 | 0 | 0 | 0.149793 | 0.221739 |
| g__Butyricimonas | 0.070847 | 0.04366 | 0.00636 | 0.011016 | 0.122287 | 0.211807 |
| g__Halomonas | 0.120387 | 0.147263 | 0 | 0 | 0.07902 | 0.105216 |
| g__Neglecta | 0.088647 | 0.06451 | 0.01676 | 0.015266 | 0.083063 | 0.098212 |
| g__GGB31256 | 0.06083 | 0.066836 | 0.05075 | 0.025401 | 0.076823 | 0.075961 |
| g__Parabacteroides | 0.0367 | 0.02861 | 0.079247 | 0.098649 | 0.066447 | 0.104792 |
| g__GGB28870 | 0.06742 | 0.115651 | 0.06861 | 0.056312 | 0.038297 | 0.026644 |
| g__GGB28766 | 0.052163 | 0.067014 | 0 | 0 | 0.12029 | 0.208063 |
| g__Pseudoflavonifractor | 0.0292 | 0.00532 | 0.09334 | 0.061034 | 0.045363 | 0.012091 |
| g__GGB31312 | 0 | 0 | 0 | 0 | 0.16622 | 0.281062 |
| g__GGB30303 | 0.097677 | 0.153959 | 0.004683 | 0.004952 | 0.063843 | 0.091252 |
| g__GGB28422 | 0.01704 | 0.029514 | 0.002613 | 0.003577 | 0.14612 | 0.153554 |
| g__GGB30297 | 0 | 0 | 0.097717 | 0.106 | 0.067803 | 0.072949 |
| g__GGB31836 | 0 | 0 | 0 | 0 | 0.164863 | 0.285552 |
| g__GGB28948 | 0.01665 | 0.014837 | 0.122957 | 0.212967 | 0.025163 | 0.022698 |
| g__GGB30147 | 0 | 0 | 0.159367 | 0.26386 | 0.001033 | 0.00179 |
| g__GGB31850 | 0 | 0 | 0 | 0 | 0.156003 | 0.251987 |
| g__Streptococcus | 0.017087 | 0.029595 | 0.138077 | 0.219581 | 0 | 0 |
| g__GGB28382 | 0.00185 | 0.003204 | 0.007253 | 0.012399 | 0.140033 | 0.242545 |
| g__GGB30457 | 0.07044 | 0.077296 | 0.06767 | 0.086839 | 0.00777 | 0.001885 |
| g__GGB28963 | 0.11536 | 0.163752 | 0.014037 | 0.011322 | 0.0154 | 0.026674 |
| g__GGB28852 | 0.00282 | 0.003452 | 0 | 0 | 0.139537 | 0.139791 |
| g__GGB28881 | 0.015443 | 0.013697 | 0.000337 | 0.000583 | 0.12109 | 0.122085 |
| g__GGB28873 | 0 | 0 | 0 | 0 | 0.136047 | 0.23564 |
| g__GGB3204 | 0.009557 | 0.016553 | 0.001773 | 0.003072 | 0.123307 | 0.193445 |
| g__GGB28403 | 0.07347 | 0.100617 | 0 | 0 | 0.059043 | 0.088535 |
| g__GGB30451 | 0.059687 | 0.05122 | 0 | 0 | 0.071733 | 0.073075 |
| g__GGB28632 | 0.063093 | 0.07599 | 0.018137 | 0.016721 | 0.04553 | 0.075125 |
| g__GGB30286 | 0.123553 | 0.108234 | 0.00048 | 0.000831 | 0.000443 | 0.000768 |
| g__GGB28846 | 0.049687 | 0.04443 | 0.042933 | 0.015938 | 0.0279 | 0.020407 |
| g__GGB28621 | 0 | 0 | 0 | 0 | 0.11821 | 0.204746 |
| g__GGB28437 | 0 | 0 | 0.070173 | 0.121544 | 0.047097 | 0.046622 |
| g__GGB28265 | 0.031337 | 0.042207 | 0.01086 | 0.009446 | 0.073467 | 0.04524 |
| g__Candidatus_Arthromitus | 0.033533 | 0.058081 | 0.044457 | 0.077001 | 0.037647 | 0.065206 |
| g__GGB30444 | 0.02888 | 0.045342 | 0.072163 | 0.048132 | 0.01284 | 0.014948 |
| g__GGB28391 | 0.010597 | 0.018354 | 0.000607 | 0.001051 | 0.10188 | 0.167378 |
| g__GGB28402 | 0.087867 | 0.105423 | 0 | 0 | 0.022277 | 0.032155 |
| g__GGB30413 | 0.049873 | 0.030284 | 0.042987 | 0.059541 | 0.013707 | 0.011954 |
| g__GGB28888 | 0 | 0 | 0 | 0 | 0.10612 | 0.122233 |
| g__GGB28379 | 0 | 0 | 0 | 0 | 0.105533 | 0.126818 |
| g__GGB28835 | 0.006177 | 0.010698 | 0 | 0 | 0.097923 | 0.169608 |
| g__GGB28869 | 0.091443 | 0.109937 | 0.01095 | 0.008283 | 0 | 0 |
| g__GGB28924 | 0 | 0 | 0.100887 | 0.101 | 0 | 0 |
| g__Acutalibacter | 0.03835 | 0.032824 | 0.021097 | 0.03654 | 0.040557 | 0.06406 |
| g__GGB28418 | 0 | 0 | 0.000177 | 0.000306 | 0.097417 | 0.142776 |
| g__GGB28779 | 0.02515 | 0.025391 | 0.006397 | 0.011079 | 0.06576 | 0.1139 |
| g__GGB28849 | 0.03193 | 0.037946 | 0.048963 | 0.08434 | 0.013973 | 0.003353 |
| g__Firmicutes_unclassified | 0.02985 | 0.051702 | 0.000243 | 0.000211 | 0.063623 | 0.106095 |
| g__Eubacterium | 0.09314 | 0.154229 | 0 | 0 | 0.000507 | 0.000878 |
| g__GGB28803 | 0.055447 | 0.05525 | 0.000227 | 0.000393 | 0.03472 | 0.033026 |
| g__GGB30091 | 0.002153 | 0.000868 | 0.058337 | 0.101042 | 0.026267 | 0.040468 |
| g__GGB28386 | 0 | 0 | 0.08546 | 0.148021 | 0.000443 | 0.000768 |
| g__GGB28405 | 0.024977 | 0.042034 | 0 | 0 | 0.06055 | 0.048624 |
| g__GGB23844 | 0.033047 | 0.049379 | 0.02221 | 0.038469 | 0.025753 | 0.027233 |
| g__GGB30301 | 0.008807 | 0.015254 | 0.007253 | 0.006449 | 0.061273 | 0.102389 |
| g__GGB30461 | 0.001557 | 0.002696 | 0.013093 | 0.01161 | 0.062627 | 0.060263 |
| g__GGB28828 | 0 | 0 | 0 | 0 | 0.07441 | 0.128882 |
| g__GGB31835 | 0 | 0 | 0 | 0 | 0.070497 | 0.1193 |
| g__GGB31841 | 0.02344 | 0.040599 | 0.037437 | 0.063708 | 0.007917 | 0.005737 |
| g__GGB28897 | 0.00202 | 0.002946 | 0.028953 | 0.038824 | 0.03558 | 0.031692 |
| g__Alphaproteobacteria_unclassified | 0.019483 | 0.033746 | 0 | 0 | 0.046043 | 0.045568 |
| g__GGB28645 | 0.000743 | 0.000648 | 0.03527 | 0.059847 | 0.027843 | 0.031023 |
| g__GGB3247 | 0.017157 | 0.029018 | 0 | 0 | 0.04635 | 0.042732 |
| g__GGB29668 | 0.043173 | 0.037854 | 0 | 0 | 0.017937 | 0.008879 |
| g__Erysipelatoclostridium | 0.002047 | 0.003545 | 0.058383 | 0.03443 | 0 | 0 |
| g__GGB28908 | 0.005577 | 0.005038 | 0 | 0 | 0.053047 | 0.09188 |
| g__GGB28695 | 0.00151 | 0.002615 | 0.05583 | 0.046671 | 0 | 0 |
| g__GGB28984 | 0 | 0 | 0 | 0 | 0.055253 | 0.092953 |
| g__GGB30290 | 0 | 0 | 0.040557 | 0.037612 | 0.01387 | 0.024024 |
| g__GGB28872 | 0.053107 | 0.091983 | 0 | 0 | 0 | 0 |
| g__GGB28408 | 0.016133 | 0.027944 | 0.016613 | 0.006915 | 0.01883 | 0.032615 |
| g__GGB29468 | 0 | 0 | 0.013307 | 0.009462 | 0.036407 | 0.036642 |
| g__GGB29006 | 0.028577 | 0.027783 | 0.004153 | 0.007194 | 0.01665 | 0.01442 |
| g__GGB31171 | 0.016273 | 0.007342 | 0.02636 | 0.001142 | 0.006727 | 0.006376 |
| g__Clostridia_unclassified | 0 | 0 | 0.046903 | 0.042477 | 0 | 0 |
| g__Flavobacteriales_unclassified | 0.046283 | 0.062746 | 8.33E-05 | 0.000144 | 0 | 0 |
| g__GGB28880 | 0.01054 | 0.018256 | 0.017977 | 0.031137 | 0.017097 | 0.027881 |
| g__GGB30093 | 0.0015 | 0.002598 | 0.007477 | 0.009704 | 0.036487 | 0.063197 |
| g__Oscillibacter | 0 | 0 | 0.045283 | 0.06405 | 0 | 0 |
| g__GGB29001 | 0.00198 | 0.003429 | 0 | 0 | 0.04324 | 0.06643 |
| g__GGB28821 | 0.02954 | 0.050853 | 0 | 0 | 0.015147 | 0.02327 |
| g__GGB28925 | 0.001313 | 0.002275 | 0.00349 | 0.006045 | 0.039483 | 0.04594 |
| g__GGB28991 | 0.00057 | 0.000987 | 0.02166 | 0.018828 | 0.017587 | 0.030461 |
| g__GGB29003 | 0 | 0 | 0 | 0 | 0.039573 | 0.054472 |
| g__GGB28960 | 0.039557 | 0.068514 | 0 | 0 | 0 | 0 |
| g__GGB28868 | 0.029837 | 0.028062 | 0 | 0 | 0.009703 | 0.010008 |
| g__GGB30466 | 0.0011 | 0.001905 | 0.017507 | 0.030322 | 0.019693 | 0.03311 |
| g__GGB28394 | 0 | 0 | 0 | 0 | 0.03665 | 0.058076 |
| g__GGB28996 | 0.000117 | 0.000202 | 0.03494 | 0.05851 | 0.001567 | 0.002714 |
| g__GGB28784 | 0.010763 | 0.003188 | 0.00322 | 0.005577 | 0.02195 | 0.007174 |
| g__GGB28883 | 0.00153 | 0.00265 | 0 | 0 | 0.034387 | 0.055471 |
| g__GGB28956 | 0.001807 | 0.003129 | 0 | 0 | 0.033653 | 0.055925 |
| g__GGB30142 | 0 | 0 | 0 | 0 | 0.03511 | 0.05768 |
| g__GGB28772 | 0.00381 | 0.003489 | 0.02106 | 0.018671 | 0.008557 | 0.014777 |
| g__GGB30448 | 0 | 0 | 0 | 0 | 0.032123 | 0.055639 |
| g__GGB28831 | 0.009093 | 0.009504 | 0.01168 | 0.016452 | 0.0104 | 0.011633 |
| g__GGB29530 | 0 | 0 | 0.029183 | 0.028641 | 0 | 0 |
| g__GGB28993 | 0 | 0 | 0.0217 | 0.037586 | 0.00645 | 0.00578 |
| g__GGB28968 | 0.005167 | 0.006055 | 0.015327 | 0.024666 | 0.006547 | 0.004037 |
| g__GGB30449 | 0 | 0 | 0.026887 | 0.018876 | 0 | 0 |
| g__GGB31851 | 0.005327 | 0.005575 | 0.002413 | 0.00418 | 0.018377 | 0.030182 |
| g__GGB30408 | 0.007383 | 0.010879 | 0.00123 | 0.00121 | 0.01739 | 0.026151 |
| g__GGB42598 | 0.0239 | 0.032103 | 0 | 0 | 0 | 0 |
| g__GGB30300 | 0.021913 | 0.018981 | 0 | 0 | 0 | 0 |
| g__GGB28887 | 0.009493 | 0.016443 | 0.001357 | 0.001579 | 0.011003 | 0.017741 |
| g__GGB28926 | 0 | 0 | 0.004847 | 0.008395 | 0.016583 | 0.028723 |
| g__GGB28415 | 0 | 0 | 0.00393 | 0.006807 | 0.017357 | 0.030063 |

Table S2. The significant enriched enzymes in fecal metagenomes between groups of DSS and HA

| Gene Family | statistic | p | method |
| --- | --- | --- | --- |
| K00003: homoserine dehydrogenase [EC:1.1.1.3]\|unclassified | 6.712644 | 0.0349 | Kruskal-Wallis |
| K00041: tagaturonate reductase [EC:1.1.1.58] | 6.488889 | 0.039 | Kruskal-Wallis |
| K00048: lactaldehyde reductase [EC:1.1.1.77]\|unclassified | 6.005602 | 0.0496 | Kruskal-Wallis |
| K00052: 3-isopropylmalate dehydrogenase [EC:1.1.1.85]\|unclassified | 6.488889 | 0.039 | Kruskal-Wallis |
| K00058: D-3-phosphoglycerate dehydrogenase [EC:1.1.1.95]\|unclassified | 6.488889 | 0.039 | Kruskal-Wallis |
| K00111: glycerol-3-phosphate dehydrogenase [EC:1.1.5.3]\|unclassified | 6.488889 | 0.039 | Kruskal-Wallis |
| K00145: N-acetyl-gamma-glutamyl-phosphate reductase [EC:1.2.1.38]\|unclassified | 6.488889 | 0.039 | Kruskal-Wallis |
| K00259: alanine dehydrogenase [EC:1.4.1.1] | 6.488889 | 0.039 | Kruskal-Wallis |
| K00370: nitrate reductase / nitrite oxidoreductase, alpha subunit [EC:1.7.5.1 1.7.99.4] | 6.72 | 0.0347 | Kruskal-Wallis |
| K00374: nitrate reductase gamma subunit [EC:1.7.5.1 1.7.99.4] | 6.763636 | 0.034 | Kruskal-Wallis |
| K00459: nitronate monooxygenase [EC:1.13.12.16] | 6.72 | 0.0347 | Kruskal-Wallis |
| K00523: CDP-4-dehydro-6-deoxyglucose reductase, E3 [EC:1.17.1.1] | 7.623529 | 0.0221 | Kruskal-Wallis |
| K00554: tRNA (guanine-N1-)-methyltransferase\|unclassified | 6.488889 | 0.039 | Kruskal-Wallis |
| K00567: methylated-DNA-[protein]-cysteine S-methyltransferase\|unclassified | 6.72 | 0.0347 | Kruskal-Wallis |
| K00571: site-specific DNA-methyltransferase (adenine-specific)\|unclassified | 6.16092 | 0.0459 | Kruskal-Wallis |
| K00602: phosphoribosylaminoimidazolecarboxamide formyltransferase / IMP cyclohydrolase [EC:2.1.2.3 3.5.4.10]\|unclassified | 7.2 | 0.0273 | Kruskal-Wallis |
| K00611: ornithine carbamoyltransferase [EC:2.1.3.3]\|unclassified | 6.488889 | 0.039 | Kruskal-Wallis |
| K00626: acetyl-CoA C-acetyltransferase [EC:2.3.1.9]\|unclassified | 7.2 | 0.0273 | Kruskal-Wallis |
| K00651: homoserine O-succinyltransferase [EC:2.3.1.46]\|unclassified | 6.488889 | 0.039 | Kruskal-Wallis |
| K00655: 1-acyl-sn-glycerol-3-phosphate acyltransferase [EC:2.3.1.51]\|unclassified | 6.543417 | 0.0379 | Kruskal-Wallis |
| K00662: aminoglycoside N3'-acetyltransferase | 6.16092 | 0.0459 | Kruskal-Wallis |
| K00694: cellulose synthase (UDP-forming) [EC:2.4.1.12] | 6.763636 | 0.034 | Kruskal-Wallis |
| K00728: dolichyl-phosphate-mannose-protein mannosyltransferase [EC:2.4.1.109]\|unclassified | 6.72 | 0.0347 | Kruskal-Wallis |
| K00773: queuine tRNA-ribosyltransferase\|unclassified | 6.488889 | 0.039 | Kruskal-Wallis |
| K00783: hypothetical protein | 6.488889 | 0.039 | Kruskal-Wallis |
| K00790: UDP-N-acetylglucosamine 1-carboxyvinyltransferase [EC:2.5.1.7]\|unclassified | 6.488889 | 0.039 | Kruskal-Wallis |
| K00794: 6,7-dimethyl-8-ribityllumazine synthase [EC:2.5.1.78]\|unclassified | 6.488889 | 0.039 | Kruskal-Wallis |
| K00805: heptaprenyl diphosphate synthase [EC:2.5.1.30]\|unclassified | 6.543417 | 0.0379 | Kruskal-Wallis |
| K00821: acetylornithine/N-succinyldiaminopimelate aminotransferase [EC:2.6.1.11 2.6.1.17]\|unclassified | 6.763636 | 0.034 | Kruskal-Wallis |
| K00831: phosphoserine aminotransferase [EC:2.6.1.52] | 6.488889 | 0.039 | Kruskal-Wallis |
| K00832: aromatic-amino-acid transaminase [EC:2.6.1.57]\|unclassified | 7.623529 | 0.0221 | Kruskal-Wallis |
| K00864: glycerol kinase [EC:2.7.1.30]\|unclassified | 7.2 | 0.0273 | Kruskal-Wallis |
| K00872: homoserine kinase [EC:2.7.1.39]\|unclassified | 6.763636 | 0.034 | Kruskal-Wallis |
| K00882: 1-phosphofructokinase [EC:2.7.1.56] | 6.488889 | 0.039 | Kruskal-Wallis |
| K00891: shikimate kinase [EC:2.7.1.71]\|unclassified | 6.488889 | 0.039 | Kruskal-Wallis |
| K00925: acetate kinase [EC:2.7.2.1]\|unclassified | 7.2 | 0.0273 | Kruskal-Wallis |
| K00926: carbamate kinase [EC:2.7.2.2]\|unclassified | 6.488889 | 0.039 | Kruskal-Wallis |
| K00930: acetylglutamate kinase [EC:2.7.2.8]\|unclassified | 6.488889 | 0.039 | Kruskal-Wallis |
| K00942: guanylate kinase [EC:2.7.4.8]\|unclassified | 6.488889 | 0.039 | Kruskal-Wallis |
| K00949: thiamine pyrophosphokinase [EC:2.7.6.2]\|unclassified | 6.005602 | 0.0496 | Kruskal-Wallis |
| K00954: pantetheine-phosphate adenylyltransferase [EC:2.7.7.3]\|unclassified | 6.488889 | 0.039 | Kruskal-Wallis |
| K00963: UTP--glucose-1-phosphate uridylyltransferase [EC:2.7.7.9]\|unclassified | 6.488889 | 0.039 | Kruskal-Wallis |
| K00971: mannose-1-phosphate guanylyltransferase [EC:2.7.7.13]\|unclassified | 6.488889 | 0.039 | Kruskal-Wallis |
| K00980: glycerol-3-phosphate cytidylyltransferase [EC:2.7.7.39] | 6.16092 | 0.0459 | Kruskal-Wallis |
| K00986: RNA-directed DNA polymerase\|unclassified | 6.543417 | 0.0379 | Kruskal-Wallis |
| K00989: ribonuclease PH | 6.488889 | 0.039 | Kruskal-Wallis |
| K01035: acetate CoA/acetoacetate CoA-transferase beta subunit [EC:2.8.3.8 2.8.3.9] | 7.623529 | 0.0221 | Kruskal-Wallis |
| K01035: acetate CoA/acetoacetate CoA-transferase beta subunit [EC:2.8.3.8 2.8.3.9]\|unclassified | 7.623529 | 0.0221 | Kruskal-Wallis |
| K01056: peptidyl-tRNA hydrolase, PTH1 family\|unclassified | 7.2 | 0.0273 | Kruskal-Wallis |
| K01057: 6-phosphogluconolactonase [EC:3.1.1.31] | 7.623529 | 0.0221 | Kruskal-Wallis |
| K01061: carboxymethylenebutenolidase [EC:3.1.1.45]\|unclassified | 7.623529 | 0.0221 | Kruskal-Wallis |
| K01066: esterase / lipase\|unclassified | 6.72 | 0.0347 | Kruskal-Wallis |
| K01070: S-formylglutathione hydrolase [EC:3.1.2.12] | 6.72 | 0.0347 | Kruskal-Wallis |
| K01095: phosphatidylglycerophosphatase A [EC:3.1.3.27] | 6.16092 | 0.0459 | Kruskal-Wallis |
| K01141: exodeoxyribonuclease I [EC:3.1.11.1] | 6.72 | 0.0347 | Kruskal-Wallis |
| K01179: endoglucanase [EC:3.2.1.4]\|unclassified | 6.72 | 0.0347 | Kruskal-Wallis |
| K01183: chitinase [EC:3.2.1.14]\|unclassified | 7.623529 | 0.0221 | Kruskal-Wallis |
| K01209: alpha-N-arabinofuranosidase [EC:3.2.1.55]\|unclassified | 6.488889 | 0.039 | Kruskal-Wallis |
| K01214: isoamylase\|unclassified | 7.623529 | 0.0221 | Kruskal-Wallis |
| K01218: mannan endo-1,4-beta-mannosidase [EC:3.2.1.78] | 6.72 | 0.0347 | Kruskal-Wallis |
| K01218: mannan endo-1,4-beta-mannosidase [EC:3.2.1.78]\|unclassified | 6.72 | 0.0347 | Kruskal-Wallis |
| K01223: 6-phospho-beta-glucosidase [EC:3.2.1.86] | 6.005602 | 0.0496 | Kruskal-Wallis |
| K01235: alpha-glucuronidase | 7.623529 | 0.0221 | Kruskal-Wallis |
| K01304: pyroglutamyl-peptidase | 6.005602 | 0.0496 | Kruskal-Wallis |
| K01358: ATP-dependent Clp protease, protease subunit [EC:3.4.21.92]\|unclassified | 7.2 | 0.0273 | Kruskal-Wallis |
| K01372: bleomycin hydrolase\|unclassified | 6.488889 | 0.039 | Kruskal-Wallis |
| K01430: urease subunit gamma [EC:3.5.1.5]\|unclassified | 6.763636 | 0.034 | Kruskal-Wallis |
| K01462: NO_NAME\|unclassified | 6.488889 | 0.039 | Kruskal-Wallis |
| K01465: dihydroorotase [EC:3.5.2.3]\|unclassified | 7.623529 | 0.0221 | Kruskal-Wallis |
| K01480: agmatinase [EC:3.5.3.11] | 7.2 | 0.0273 | Kruskal-Wallis |
| K01487: guanine deaminase [EC:3.5.4.3]\|unclassified | 6.72 | 0.0347 | Kruskal-Wallis |
| K01496: phosphoribosyl-AMP cyclohydrolase [EC:3.5.4.19]\|unclassified | 7.623529 | 0.0221 | Kruskal-Wallis |
| K01523: phosphoribosyl-ATP pyrophosphohydrolase [EC:3.6.1.31]\|unclassified | 7.623529 | 0.0221 | Kruskal-Wallis |
| K01524: exopolyphosphatase / guanosine-5'-triphosphate,3'-diphosphate pyrophosphatase [EC:3.6.1.11 3.6.1.40]\|unclassified | 6.72 | 0.0347 | Kruskal-Wallis |
| K01537: Ca2+-transporting ATPase | 6.488889 | 0.039 | Kruskal-Wallis |
| K01547: K+-transporting ATPase ATPase B chain [EC:3.6.3.12] | 7.623529 | 0.0221 | Kruskal-Wallis |
| K01560: 2-haloacid dehalogenase [EC:3.8.1.2]\|unclassified | 7.623529 | 0.0221 | Kruskal-Wallis |
| K01581: ornithine decarboxylase [EC:4.1.1.17]\|unclassified | 7.623529 | 0.0221 | Kruskal-Wallis |
| K01596: phosphoenolpyruvate carboxykinase (GTP) [EC:4.1.1.32]\|unclassified | 6.72 | 0.0347 | Kruskal-Wallis |
| K01619: deoxyribose-phosphate aldolase [EC:4.1.2.4] | 6.488889 | 0.039 | Kruskal-Wallis |
| K01629: rhamnulose-1-phosphate aldolase [EC:4.1.2.19]\|unclassified | 6.543417 | 0.0379 | Kruskal-Wallis |
| K01679: fumarate hydratase, class II [EC:4.2.1.2] | 6.543417 | 0.0379 | Kruskal-Wallis |
| K01685: altronate hydrolase [EC:4.2.1.7]\|unclassified | 6.488889 | 0.039 | Kruskal-Wallis |
| K01703: 3-isopropylmalate/(R)-2-methylmalate dehydratase large subunit [EC:4.2.1.33 4.2.1.35]\|unclassified | 6.488889 | 0.039 | Kruskal-Wallis |
| K01715: enoyl-CoA hydratase [EC:4.2.1.17]\|unclassified | 7.623529 | 0.0221 | Kruskal-Wallis |
| K01734: methylglyoxal synthase [EC:4.2.3.3]\|unclassified | 7.2 | 0.0273 | Kruskal-Wallis |
| K01745: histidine ammonia-lyase [EC:4.3.1.3]\|unclassified | 6.488889 | 0.039 | Kruskal-Wallis |
| K01751: diaminopropionate ammonia-lyase\|unclassified | 7.260504 | 0.0265 | Kruskal-Wallis |
| K01785: aldose 1-epimerase [EC:5.1.3.3]\|unclassified | 7.2 | 0.0273 | Kruskal-Wallis |
| K01804: L-arabinose isomerase [EC:5.3.1.4]\|unclassified | 7.2 | 0.0273 | Kruskal-Wallis |
| K01810: glucose-6-phosphate isomerase [EC:5.3.1.9]\|unclassified | 6.488889 | 0.039 | Kruskal-Wallis |
| K01811: putative family 31 glucosidase\|unclassified | 6.543417 | 0.0379 | Kruskal-Wallis |
| K01814: phosphoribosylformimino-5-aminoimidazole carboxamide ribotide isomerase [EC:5.3.1.16]\|unclassified | 6.005602 | 0.0496 | Kruskal-Wallis |
| K01815: 4-deoxy-L-threo-5-hexosulose-uronate ketol-isomerase [EC:5.3.1.17]\|unclassified | 6.488889 | 0.039 | Kruskal-Wallis |
| K01844: beta-lysine 5,6-aminomutase alpha subunit [EC:5.4.3.3] | 6.72 | 0.0347 | Kruskal-Wallis |
| K01844: beta-lysine 5,6-aminomutase alpha subunit [EC:5.4.3.3]\|unclassified | 6.72 | 0.0347 | Kruskal-Wallis |
| K01866: tyrosyl-tRNA synthetase [EC:6.1.1.1]\|unclassified | 7.2 | 0.0273 | Kruskal-Wallis |
| K01872: alanyl-tRNA synthetase [EC:6.1.1.7]\|unclassified | 6.488889 | 0.039 | Kruskal-Wallis |
| K01873: valyl-tRNA synthetase [EC:6.1.1.9]\|unclassified | 6.488889 | 0.039 | Kruskal-Wallis |
| K01875: seryl-tRNA synthetase [EC:6.1.1.11]\|unclassified | 6.488889 | 0.039 | Kruskal-Wallis |
| K01883: cysteinyl-tRNA synthetase [EC:6.1.1.16]\|unclassified | 7.2 | 0.0273 | Kruskal-Wallis |
| K01890: phenylalanyl-tRNA synthetase beta chain [EC:6.1.1.20]\|unclassified | 7.2 | 0.0273 | Kruskal-Wallis |
| K01892: histidyl-tRNA synthetase [EC:6.1.1.21]\|unclassified | 6.488889 | 0.039 | Kruskal-Wallis |
| K01912: phenylacetate-CoA ligase [EC:6.2.1.30]\|unclassified | 6.488889 | 0.039 | Kruskal-Wallis |
| K01918: pantoate--beta-alanine ligase [EC:6.3.2.1]\|unclassified | 7.2 | 0.0273 | Kruskal-Wallis |
| K01921: D-alanine-D-alanine ligase [EC:6.3.2.4] | 7.2 | 0.0273 | Kruskal-Wallis |
| K01938: formate--tetrahydrofolate ligase [EC:6.3.4.3]\|unclassified | 6.488889 | 0.039 | Kruskal-Wallis |
| K01972: DNA ligase (NAD+) [EC:6.5.1.2]\|unclassified | 6.488889 | 0.039 | Kruskal-Wallis |
| K01990: ABC-2 type transport system ATP-binding protein\|unclassified | 7.2 | 0.0273 | Kruskal-Wallis |
| K01991: polysaccharide export outer membrane protein | 6.763636 | 0.034 | Kruskal-Wallis |
| K01995: branched-chain amino acid transport system ATP-binding protein | 6.488889 | 0.039 | Kruskal-Wallis |
| K01995: branched-chain amino acid transport system ATP-binding protein\|unclassified | 7.2 | 0.0273 | Kruskal-Wallis |
| K01996: branched-chain amino acid transport system ATP-binding protein\|unclassified | 7.2 | 0.0273 | Kruskal-Wallis |
| K02009: cobalt/nickel transport protein | 7.623529 | 0.0221 | Kruskal-Wallis |
| K02011: iron(III) transport system permease protein | 7.623529 | 0.0221 | Kruskal-Wallis |
| K02012: iron(III) transport system substrate-binding protein | 7.623529 | 0.0221 | Kruskal-Wallis |
| K02013: iron complex transport system ATP-binding protein [EC:3.6.3.34]\|unclassified | 6.488889 | 0.039 | Kruskal-Wallis |
| K02014: iron complex outermembrane recepter protein\|unclassified | 6.488889 | 0.039 | Kruskal-Wallis |
| K02019: molybdate transport system regulatory protein\|unclassified | 6.763636 | 0.034 | Kruskal-Wallis |
| K02029: polar amino acid transport system permease protein\|unclassified | 7.2 | 0.0273 | Kruskal-Wallis |
| K02052: putative spermidine/putrescine transport system ATP-binding protein | 7.623529 | 0.0221 | Kruskal-Wallis |
| K02054: putative spermidine/putrescine transport system permease protein\|unclassified | 7.623529 | 0.0221 | Kruskal-Wallis |
| K02055: putative spermidine/putrescine transport system substrate-binding protein | 7.623529 | 0.0221 | Kruskal-Wallis |
| K02066: phospholipid/cholesterol/gamma-HCH transport system permease protein\|unclassified | 7.2 | 0.0273 | Kruskal-Wallis |
| K02081: DeoR family transcriptional regulator, aga operon transcriptional\|unclassified | 7.623529 | 0.0221 | Kruskal-Wallis |
| K02112: F-type H+-transporting ATPase subunit beta [EC:3.6.3.14]\|unclassified | 7.2 | 0.0273 | Kruskal-Wallis |
| K02193: heme exporter protein A [EC:3.6.3.41] | 6.72 | 0.0347 | Kruskal-Wallis |
| K02196: heme exporter protein D | 6.16092 | 0.0459 | Kruskal-Wallis |
| K02227: adenosylcobinamide-phosphate synthase [EC:6.3.1.10] | 7.623529 | 0.0221 | Kruskal-Wallis |
| K02227: adenosylcobinamide-phosphate synthase [EC:6.3.1.10]\|unclassified | 7.623529 | 0.0221 | Kruskal-Wallis |
| K02316: DNA primase [EC:2.7.7.-] | 7.2 | 0.0273 | Kruskal-Wallis |
| K02334: DNA polymerase bacteriophage-type | 7.2 | 0.0273 | Kruskal-Wallis |
| K02334: DNA polymerase bacteriophage-type\|unclassified | 6.488889 | 0.039 | Kruskal-Wallis |
| K02358: elongation factor EF-Tu\|unclassified | 6.488889 | 0.039 | Kruskal-Wallis |
| K02387: flagellar basal-body rod protein FlgB\|unclassified | 7.623529 | 0.0221 | Kruskal-Wallis |
| K02401: flagellar biosynthetic protein FlhB\|unclassified | 7.623529 | 0.0221 | Kruskal-Wallis |
| K02405: RNA polymerase sigma factor for flagellar operon FliA | 6.488889 | 0.039 | Kruskal-Wallis |
| K02405: RNA polymerase sigma factor for flagellar operon FliA\|unclassified | 7.260504 | 0.0265 | Kruskal-Wallis |
| K02406: flagellin | 6.543417 | 0.0379 | Kruskal-Wallis |
| K02406: flagellin\|unclassified | 6.543417 | 0.0379 | Kruskal-Wallis |
| K02410: flagellar motor switch protein FliG\|unclassified | 6.488889 | 0.039 | Kruskal-Wallis |
| K02412: flagellum-specific ATP synthase [EC:3.6.3.14] | 6.488889 | 0.039 | Kruskal-Wallis |
| K02412: flagellum-specific ATP synthase [EC:3.6.3.14]\|unclassified | 6.488889 | 0.039 | Kruskal-Wallis |
| K02420: flagellar biosynthetic protein FliQ | 6.488889 | 0.039 | Kruskal-Wallis |
| K02420: flagellar biosynthetic protein FliQ\|unclassified | 6.488889 | 0.039 | Kruskal-Wallis |
| K02473: UDP-N-acetylglucosamine 4-epimerase [EC:5.1.3.7] | 7.623529 | 0.0221 | Kruskal-Wallis |
| K02473: UDP-N-acetylglucosamine 4-epimerase [EC:5.1.3.7]\|unclassified | 7.623529 | 0.0221 | Kruskal-Wallis |
| K02484: two-component system, OmpR family, sensor kinase | 6.72 | 0.0347 | Kruskal-Wallis |
| K02494: outer membrane lipoprotein LolB | 6.72 | 0.0347 | Kruskal-Wallis |
| K02499: tetrapyrrole methylase family protein / MazG family protein | 6.16092 | 0.0459 | Kruskal-Wallis |
| K02499: tetrapyrrole methylase family protein / MazG family protein\|unclassified | 6.16092 | 0.0459 | Kruskal-Wallis |
| K02500: cyclase [EC:4.1.3.-]\|unclassified | 6.488889 | 0.039 | Kruskal-Wallis |
| K02502: ATP phosphoribosyltransferase regulatory subunit\|unclassified | 7.623529 | 0.0221 | Kruskal-Wallis |
| K02556: chemotaxis protein MotA | 7.260504 | 0.0265 | Kruskal-Wallis |
| K02556: chemotaxis protein MotA\|unclassified | 6.543417 | 0.0379 | Kruskal-Wallis |
| K02558: UDP-N-acetylmuramate: L-alanyl-gamma-D-glutamyl-meso-diaminopimelate | 6.72 | 0.0347 | Kruskal-Wallis |
| K02567: periplasmic nitrate reductase NapA [EC:1.7.99.4]\|unclassified | 7.623529 | 0.0221 | Kruskal-Wallis |
| K02573: ferredoxin-type protein NapG | 6.712644 | 0.0349 | Kruskal-Wallis |
| K02759: PTS system, cellobiose-specific IIA component [EC:2.7.1.196 2.7.1.205] | 6.763636 | 0.034 | Kruskal-Wallis |
| K02793: PTS system, mannose-specific IIA component [EC:2.7.1.191] | 6.005602 | 0.0496 | Kruskal-Wallis |
| K02825: pyrimidine operon attenuation protein / uracil phosphoribosyltransferase [EC:2.4.2.9] | 6.488889 | 0.039 | Kruskal-Wallis |
| K02836: peptide chain release factor RF-2 | 6.488889 | 0.039 | Kruskal-Wallis |
| K02851: undecaprenyl-phosphate alpha-N-acetylglucosaminyltransferase\|unclassified | 6.72 | 0.0347 | Kruskal-Wallis |
| K02867: large subunit ribosomal protein L11\|unclassified | 7.2 | 0.0273 | Kruskal-Wallis |
| K02884: large subunit ribosomal protein L19 | 6.488889 | 0.039 | Kruskal-Wallis |
| K02886: large subunit ribosomal protein L2\|unclassified | 7.2 | 0.0273 | Kruskal-Wallis |
| K02892: large subunit ribosomal protein L23\|unclassified | 7.2 | 0.0273 | Kruskal-Wallis |
| K02904: large subunit ribosomal protein L29\|unclassified | 7.2 | 0.0273 | Kruskal-Wallis |
| K02909: large subunit ribosomal protein L31\|unclassified | 7.2 | 0.0273 | Kruskal-Wallis |
| K02914: large subunit ribosomal protein L34 | 7.2 | 0.0273 | Kruskal-Wallis |
| K02933: large subunit ribosomal protein L6\|unclassified | 7.2 | 0.0273 | Kruskal-Wallis |
| K02939: large subunit ribosomal protein L9\|unclassified | 6.488889 | 0.039 | Kruskal-Wallis |
| K02946: small subunit ribosomal protein S10\|unclassified | 6.488889 | 0.039 | Kruskal-Wallis |
| K02950: small subunit ribosomal protein S12\|unclassified | 6.488889 | 0.039 | Kruskal-Wallis |
| K02956: small subunit ribosomal protein S15\|unclassified | 7.2 | 0.0273 | Kruskal-Wallis |
| K02967: small subunit ribosomal protein S2\|unclassified | 6.488889 | 0.039 | Kruskal-Wallis |
| K02970: small subunit ribosomal protein S21 | 6.488889 | 0.039 | Kruskal-Wallis |
| K02970: small subunit ribosomal protein S21\|unclassified | 7.2 | 0.0273 | Kruskal-Wallis |
| K02990: small subunit ribosomal protein S6\|unclassified | 7.2 | 0.0273 | Kruskal-Wallis |
| K02992: small subunit ribosomal protein S7\|unclassified | 6.488889 | 0.039 | Kruskal-Wallis |
| K02994: small subunit ribosomal protein S8\|unclassified | 6.488889 | 0.039 | Kruskal-Wallis |
| K03060: DNA-directed RNA polymerase subunit omega [EC:2.7.7.6] | 6.488889 | 0.039 | Kruskal-Wallis |
| K03060: DNA-directed RNA polymerase subunit omega [EC:2.7.7.6]\|unclassified | 7.2 | 0.0273 | Kruskal-Wallis |
| K03072: preprotein translocase subunit SecD | 7.448276 | 0.0241 | Kruskal-Wallis |
| K03077: L-ribulose-5-phosphate 4-epimerase [EC:5.1.3.4]\|unclassified | 7.2 | 0.0273 | Kruskal-Wallis |
| K03086: RNA polymerase primary sigma factor\|unclassified | 7.2 | 0.0273 | Kruskal-Wallis |
| K03091: RNA polymerase sporulation-specific sigma factor\|unclassified | 6.488889 | 0.039 | Kruskal-Wallis |
| K03101: signal peptidase II [EC:3.4.23.36]\|unclassified | 7.260504 | 0.0265 | Kruskal-Wallis |
| K03215: RNA methyltransferase, TrmA family\|unclassified | 7.2 | 0.0273 | Kruskal-Wallis |
| K03271: D-sedoheptulose 7-phosphate isomerase [EC:5.3.1.28] | 6.488889 | 0.039 | Kruskal-Wallis |
| K03298: drug/metabolite transporter, DME family | 7.623529 | 0.0221 | Kruskal-Wallis |
| K03299: gluconate:H+ symporter, GntP family\|unclassified | 7.623529 | 0.0221 | Kruskal-Wallis |
| K03308: neurotransmitter:Na+ symporter, NSS family\|unclassified | 6.488889 | 0.039 | Kruskal-Wallis |
| K03313: Na+:H+ antiporter, NhaA family\|unclassified | 7.2 | 0.0273 | Kruskal-Wallis |
| K03326: C4-dicarboxylate transporter, DcuC family | 6.712644 | 0.0349 | Kruskal-Wallis |
| K03326: C4-dicarboxylate transporter, DcuC family\|unclassified | 6.72 | 0.0347 | Kruskal-Wallis |
| K03336: 3D-(3,5/4)-trihydroxycyclohexane-1,2-dione acylhydrolase (decyclizing) [EC:3.7.1.22] | 7.623529 | 0.0221 | Kruskal-Wallis |
| K03336: 3D-(3,5/4)-trihydroxycyclohexane-1,2-dione acylhydrolase (decyclizing) [EC:3.7.1.22]\|unclassified | 7.623529 | 0.0221 | Kruskal-Wallis |
| K03413: two-component system, chemotaxis family, response regulator CheY | 6.488889 | 0.039 | Kruskal-Wallis |
| K03413: two-component system, chemotaxis family, response regulator CheY\|unclassified | 6.488889 | 0.039 | Kruskal-Wallis |
| K03439: tRNA (guanine-N7-)-methyltransferase\|unclassified | 7.2 | 0.0273 | Kruskal-Wallis |
| K03478: hypothetical protein | 7.623529 | 0.0221 | Kruskal-Wallis |
| K03492: GntR family transcriptional regulator | 7.623529 | 0.0221 | Kruskal-Wallis |
| K03495: glucose inhibited division protein A\|unclassified | 7.2 | 0.0273 | Kruskal-Wallis |
| K03497: chromosome partitioning protein, ParB family | 6.488889 | 0.039 | Kruskal-Wallis |
| K03497: chromosome partitioning protein, ParB family\|unclassified | 6.488889 | 0.039 | Kruskal-Wallis |
| K03502: DNA polymerase V\|unclassified | 6.72 | 0.0347 | Kruskal-Wallis |
| K03526: (E)-4-hydroxy-3-methylbut-2-enyl-diphosphate synthase [EC:1.17.7.1 1.17.7.3]\|unclassified | 7.2 | 0.0273 | Kruskal-Wallis |
| K03531: cell division protein FtsZ\|unclassified | 7.2 | 0.0273 | Kruskal-Wallis |
| K03534: L-rhamnose mutarotase\|unclassified | 6.488889 | 0.039 | Kruskal-Wallis |
| K03544: ATP-dependent Clp protease ATP-binding subunit ClpX\|unclassified | 7.2 | 0.0273 | Kruskal-Wallis |
| K03553: recombination protein RecA | 6.488889 | 0.039 | Kruskal-Wallis |
| K03553: recombination protein RecA\|unclassified | 7.2 | 0.0273 | Kruskal-Wallis |
| K03568: TldD protein\|unclassified | 7.448276 | 0.0241 | Kruskal-Wallis |
| K03571: rod shape-determining protein MreD\|unclassified | 6.763636 | 0.034 | Kruskal-Wallis |
| K03578: ATP-dependent helicase HrpA | 6.72 | 0.0347 | Kruskal-Wallis |
| K03581: exodeoxyribonuclease V alpha subunit [EC:3.1.11.5]\|unclassified | 6.16092 | 0.0459 | Kruskal-Wallis |
| K03592: PmbA protein\|unclassified | 6.712644 | 0.0349 | Kruskal-Wallis |
| K03593: ATP-binding protein involved in chromosome partitioning\|unclassified | 6.16092 | 0.0459 | Kruskal-Wallis |
| K03594: bacterioferritin [EC:1.16.3.1] | 7.623529 | 0.0221 | Kruskal-Wallis |
| K03596: GTP-binding protein LepA\|unclassified | 7.2 | 0.0273 | Kruskal-Wallis |
| K03615: electron transport complex protein RnfC\|unclassified | 7.2 | 0.0273 | Kruskal-Wallis |
| K03617: electron transport complex protein RnfA\|unclassified | 7.2 | 0.0273 | Kruskal-Wallis |
| K03620: Ni/Fe-hydrogenase 1 B-type cytochrome subunit | 6.72 | 0.0347 | Kruskal-Wallis |
| K03621: glycerol-3-phosphate acyltransferase PlsX [EC:2.3.1.15]\|unclassified | 7.2 | 0.0273 | Kruskal-Wallis |
| K03624: transcription elongation factor GreA\|unclassified | 7.2 | 0.0273 | Kruskal-Wallis |
| K03636: molybdopterin synthase sulfur carrier subunit | 6.72 | 0.0347 | Kruskal-Wallis |
| K03657: DNA helicase II / ATP-dependent DNA helicase PcrA [EC:3.6.4.12]\|unclassified | 6.543417 | 0.0379 | Kruskal-Wallis |
| K03685: ribonuclease III [EC:3.1.26.3]\|unclassified | 6.488889 | 0.039 | Kruskal-Wallis |
| K03703: excinuclease ABC subunit C\|unclassified | 7.2 | 0.0273 | Kruskal-Wallis |
| K03704: cold shock protein (beta-ribbon, CspA family)\|unclassified | 7.2 | 0.0273 | Kruskal-Wallis |
| K03705: heat-inducible transcriptional repressor\|unclassified | 6.72 | 0.0347 | Kruskal-Wallis |
| K03742: nicotinamide-nucleotide amidase [EC:3.5.1.42]\|unclassified | 6.488889 | 0.039 | Kruskal-Wallis |
| K03748: SanA protein | 6.488889 | 0.039 | Kruskal-Wallis |
| K03748: SanA protein\|unclassified | 7.623529 | 0.0221 | Kruskal-Wallis |
| K03750: molybdopterin biosynthesis protein MoeA | 6.72 | 0.0347 | Kruskal-Wallis |
| K03756: putrescine:ornithine antiporter\|unclassified | 7.623529 | 0.0221 | Kruskal-Wallis |
| K03771: peptidyl-prolyl cis-trans isomerase SurA\|unclassified | 6.16092 | 0.0459 | Kruskal-Wallis |
| K03790: ribosomal-protein-alanine N-acetyltransferase\|unclassified | 7.623529 | 0.0221 | Kruskal-Wallis |
| K03797: carboxyl-terminal processing protease\|unclassified | 7.2 | 0.0273 | Kruskal-Wallis |
| K03798: cell division protease FtsH\|unclassified | 6.488889 | 0.039 | Kruskal-Wallis |
| K03801: lipoyl(octanoyl) transferase [EC:2.3.1.181]\|unclassified | 6.488889 | 0.039 | Kruskal-Wallis |
| K03805: thiol:disulfide interchange protein DsbG | 6.72 | 0.0347 | Kruskal-Wallis |
| K03811: nicotinamide mononucleotide transporter\|unclassified | 6.488889 | 0.039 | Kruskal-Wallis |
| K03880: NADH-ubiquinone oxidoreductase chain 3 [EC:1.6.5.3] | 6.488889 | 0.039 | Kruskal-Wallis |
| K03880: NADH-ubiquinone oxidoreductase chain 3 [EC:1.6.5.3]\|unclassified | 6.488889 | 0.039 | Kruskal-Wallis |
| K03882: NADH-ubiquinone oxidoreductase chain 4L [EC:1.6.5.3] | 7.2 | 0.0273 | Kruskal-Wallis |
| K03882: NADH-ubiquinone oxidoreductase chain 4L [EC:1.6.5.3]\|unclassified | 7.2 | 0.0273 | Kruskal-Wallis |
| K04015: formate-dependent nitrate reductase complex, transmembrane protein | 6.72 | 0.0347 | Kruskal-Wallis |
| K04027: ethanolamine utilization protein EutM\|unclassified | 7.623529 | 0.0221 | Kruskal-Wallis |
| K04032: ethanolamine utilization cobalamin adenosyltransferase [EC:2.5.1.17]\|unclassified | 7.623529 | 0.0221 | Kruskal-Wallis |
| K04043: molecular chaperone DnaK\|unclassified | 6.488889 | 0.039 | Kruskal-Wallis |
| K04068: anaerobic ribonucleoside-triphosphate reductase activating protein\|unclassified | 6.16092 | 0.0459 | Kruskal-Wallis |
| K04070: putative pyruvate formate lyase activating enzyme\|unclassified | 7.623529 | 0.0221 | Kruskal-Wallis |
| K04087: membrane protease subunit HflC\|unclassified | 7.2 | 0.0273 | Kruskal-Wallis |
| K04094: glucose inhibited division protein Gid | 6.543417 | 0.0379 | Kruskal-Wallis |
| K04094: glucose inhibited division protein Gid\|unclassified | 6.72 | 0.0347 | Kruskal-Wallis |
| K04096: DNA processing protein\|unclassified | 6.005602 | 0.0496 | Kruskal-Wallis |
| K04486: histidinol-phosphatase (PHP family) [EC:3.1.3.15]\|unclassified | 6.488889 | 0.039 | Kruskal-Wallis |
| K04517: prephenate dehydrogenase [EC:1.3.1.12]\|unclassified | 7.623529 | 0.0221 | Kruskal-Wallis |
| K04551: ubiquitin B | 7.623529 | 0.0221 | Kruskal-Wallis |
| K04551: ubiquitin B\|unclassified | 7.623529 | 0.0221 | Kruskal-Wallis |
| K04656: hydrogenase maturation protein HypF | 7.623529 | 0.0221 | Kruskal-Wallis |
| K04760: transcription elongation factor GreB | 6.72 | 0.0347 | Kruskal-Wallis |
| K04771: serine protease Do [EC:3.4.21.107]\|unclassified | 7.623529 | 0.0221 | Kruskal-Wallis |
| K05516: curved DNA-binding protein | 6.16092 | 0.0459 | Kruskal-Wallis |
| K05516: curved DNA-binding protein\|unclassified | 7.623529 | 0.0221 | Kruskal-Wallis |
| K05592: ATP-dependent RNA helicase DeaD [EC:3.6.4.13]\|unclassified | 6.488889 | 0.039 | Kruskal-Wallis |
| K05601: hydroxylamine reductase [EC:1.7.99.1]\|unclassified | 7.623529 | 0.0221 | Kruskal-Wallis |
| K05788: integration host factor subunit beta\|unclassified | 6.16092 | 0.0459 | Kruskal-Wallis |
| K05807: putative lipoprotein\|unclassified | 6.488889 | 0.039 | Kruskal-Wallis |
| K05810: conserved hypothetical protein\|unclassified | 7.623529 | 0.0221 | Kruskal-Wallis |
| K05813: sn-glycerol 3-phosphate transport system substrate-binding protein\|unclassified | 6.005602 | 0.0496 | Kruskal-Wallis |
| K05822: tetrahydrodipicolinate N-acetyltransferase [EC:2.3.1.89] | 6.16092 | 0.0459 | Kruskal-Wallis |
| K05825: 2-aminoadipate transaminase [EC:2.6.1.-]\|unclassified | 7.623529 | 0.0221 | Kruskal-Wallis |
| K05832: putative ABC transport system permease protein\|unclassified | 7.2 | 0.0273 | Kruskal-Wallis |
| K05946: N-acetylglucosaminyldiphosphoundecaprenol | 6.543417 | 0.0379 | Kruskal-Wallis |
| K05985: ribonuclease M5\|unclassified | 7.623529 | 0.0221 | Kruskal-Wallis |
| K05989: alpha-L-rhamnosidase\|unclassified | 6.72 | 0.0347 | Kruskal-Wallis |
| K05995: dipeptidase E\|unclassified | 7.623529 | 0.0221 | Kruskal-Wallis |
| K06012: spore protease | 6.72 | 0.0347 | Kruskal-Wallis |
| K06012: spore protease\|unclassified | 6.72 | 0.0347 | Kruskal-Wallis |
| K06013: STE24 endopeptidase [EC:3.4.24.84] | 7.623529 | 0.0221 | Kruskal-Wallis |
| K06024: segregation and condensation protein B\|unclassified | 6.72 | 0.0347 | Kruskal-Wallis |
| K06168: bifunctional enzyme involved in thiolation and methylation of tRNA\|unclassified | 7.2 | 0.0273 | Kruskal-Wallis |
| K06192: paraquat-inducible protein B | 6.72 | 0.0347 | Kruskal-Wallis |
| K06200: carbon starvation protein | 6.763636 | 0.034 | Kruskal-Wallis |
| K06200: carbon starvation protein\|unclassified | 6.763636 | 0.034 | Kruskal-Wallis |
| K06215: pyridoxal 5'-phosphate synthase pdxS subunit [EC:4.3.3.6]\|unclassified | 7.2 | 0.0273 | Kruskal-Wallis |
| K06219: S-adenosylmethionine-dependent methyltransferase\|unclassified | 7.623529 | 0.0221 | Kruskal-Wallis |
| K06295: spore germination protein KA\|unclassified | 7.623529 | 0.0221 | Kruskal-Wallis |
| K06382: stage II sporulation protein E\|unclassified | 6.72 | 0.0347 | Kruskal-Wallis |
| K06387: stage II sporulation protein R | 6.72 | 0.0347 | Kruskal-Wallis |
| K06387: stage II sporulation protein R\|unclassified | 6.72 | 0.0347 | Kruskal-Wallis |
| K06897: NO_NAME | 7.623529 | 0.0221 | Kruskal-Wallis |
| K06898: NO_NAME | 6.72 | 0.0347 | Kruskal-Wallis |
| K06902: MFS transporter, UMF1 family\|unclassified | 6.763636 | 0.034 | Kruskal-Wallis |
| K06919: NO_NAME\|unclassified | 7.2 | 0.0273 | Kruskal-Wallis |
| K06970: ribosomal RNA large subunit methyltransferase F | 7.623529 | 0.0221 | Kruskal-Wallis |
| K06987: NO_NAME | 7.623529 | 0.0221 | Kruskal-Wallis |
| K07000: NO_NAME | 6.72 | 0.0347 | Kruskal-Wallis |
| K07005: NO_NAME\|unclassified | 7.623529 | 0.0221 | Kruskal-Wallis |
| K07018: NO_NAME | 7.623529 | 0.0221 | Kruskal-Wallis |
| K07019: NO_NAME | 7.623529 | 0.0221 | Kruskal-Wallis |
| K07023: putative hydrolases of HD superfamily | 7.448276 | 0.0241 | Kruskal-Wallis |
| K07025: putative hydrolase of the HAD superfamily\|unclassified | 6.488889 | 0.039 | Kruskal-Wallis |
| K07032: NO_NAME | 6.72 | 0.0347 | Kruskal-Wallis |
| K07032: NO_NAME\|unclassified | 6.72 | 0.0347 | Kruskal-Wallis |
| K07035: NO_NAME\|unclassified | 6.72 | 0.0347 | Kruskal-Wallis |
| K07043: NO_NAME\|unclassified | 7.260504 | 0.0265 | Kruskal-Wallis |
| K07045: NO_NAME\|unclassified | 6.763636 | 0.034 | Kruskal-Wallis |
| K07053: NO_NAME\|unclassified | 6.72 | 0.0347 | Kruskal-Wallis |
| K07089: NO_NAME | 7.2 | 0.0273 | Kruskal-Wallis |
| K07089: NO_NAME\|unclassified | 7.260504 | 0.0265 | Kruskal-Wallis |
| K07090: NO_NAME\|unclassified | 7.2 | 0.0273 | Kruskal-Wallis |
| K07166: ACT domain-containing protein\|unclassified | 6.72 | 0.0347 | Kruskal-Wallis |
| K07218: nitrous oxidase accessory protein | 7.623529 | 0.0221 | Kruskal-Wallis |
| K07220: hypothetical protein | 7.623529 | 0.0221 | Kruskal-Wallis |
| K07284: sortase A | 6.543417 | 0.0379 | Kruskal-Wallis |
| K07284: sortase A\|unclassified | 6.72 | 0.0347 | Kruskal-Wallis |
| K07307: anaerobic dimethyl sulfoxide reductase subunit B (DMSO reductase iron- sulfur subunit) | 6.16092 | 0.0459 | Kruskal-Wallis |
| K07335: basic membrane protein A and related proteins | 6.763636 | 0.034 | Kruskal-Wallis |
| K07335: basic membrane protein A and related proteins\|unclassified | 7.623529 | 0.0221 | Kruskal-Wallis |
| K07391: magnesium chelatase family protein | 7.2 | 0.0273 | Kruskal-Wallis |
| K07404: 6-phosphogluconolactonase [EC:3.1.1.31]\|unclassified | 7.623529 | 0.0221 | Kruskal-Wallis |
| K07458: DNA mismatch endonuclease, patch repair protein\|unclassified | 7.2 | 0.0273 | Kruskal-Wallis |
| K07651: two-component system, OmpR family, sensor histidine kinase ResE [EC:2.7.13.3] | 7.623529 | 0.0221 | Kruskal-Wallis |
| K07699: two-component system, response regulator, stage 0 sporulation protein A\|unclassified | 6.72 | 0.0347 | Kruskal-Wallis |
| K07736: CarD family transcriptional regulator | 6.16092 | 0.0459 | Kruskal-Wallis |
| K07736: CarD family transcriptional regulator\|unclassified | 6.16092 | 0.0459 | Kruskal-Wallis |
| K07791: anaerobic C4-dicarboxylate transporter DcuA\|unclassified | 7.623529 | 0.0221 | Kruskal-Wallis |
| K07794: putative tricarboxylic transport membrane protein | 7.623529 | 0.0221 | Kruskal-Wallis |
| K08159: MFS transporter, DHA1 family, | 6.72 | 0.0347 | Kruskal-Wallis |
| K08222: MFS transporter, YQGE family, putative transporter | 7.623529 | 0.0221 | Kruskal-Wallis |
| K08281: nicotinamidase/pyrazinamidase [EC:3.5.1.19 3.5.1.-] | 6.488889 | 0.039 | Kruskal-Wallis |
| K08300: ribonuclease E [EC:3.1.26.12] | 6.72 | 0.0347 | Kruskal-Wallis |
| K08305: membrane-bound lytic murein transglycosylase B | 6.72 | 0.0347 | Kruskal-Wallis |
| K08722: 5'-deoxynucleotidase [EC:3.1.3.89]\|unclassified | 6.72 | 0.0347 | Kruskal-Wallis |
| K08968: L-methionine (R)-S-oxide reductase [EC:1.8.4.14] | 6.712644 | 0.0349 | Kruskal-Wallis |
| K08968: L-methionine (R)-S-oxide reductase [EC:1.8.4.14]\|unclassified | 6.72 | 0.0347 | Kruskal-Wallis |
| K09001: anhydro-N-acetylmuramic acid kinase | 6.72 | 0.0347 | Kruskal-Wallis |
| K09133: hypothetical protein | 7.623529 | 0.0221 | Kruskal-Wallis |
| K09157: hypothetical protein\|unclassified | 7.2 | 0.0273 | Kruskal-Wallis |
| K09181: hypothetical protein | 6.72 | 0.0347 | Kruskal-Wallis |
| K09474: acid phosphatase (class A) [EC:3.1.3.2] | 7.623529 | 0.0221 | Kruskal-Wallis |
| K09710: hypothetical protein\|unclassified | 7.623529 | 0.0221 | Kruskal-Wallis |
| K09761: ribosomal RNA small subunit methyltransferase E\|unclassified | 7.2 | 0.0273 | Kruskal-Wallis |
| K09777: hypothetical protein | 7.2 | 0.0273 | Kruskal-Wallis |
| K09777: hypothetical protein\|unclassified | 7.2 | 0.0273 | Kruskal-Wallis |
| K09806: hypothetical protein\|unclassified | 7.623529 | 0.0221 | Kruskal-Wallis |
| K09809: CDP-glycerol glycerophosphotransferase | 7.623529 | 0.0221 | Kruskal-Wallis |
| K09809: CDP-glycerol glycerophosphotransferase\|unclassified | 7.623529 | 0.0221 | Kruskal-Wallis |
| K09815: zinc transport system substrate-binding protein\|unclassified | 6.72 | 0.0347 | Kruskal-Wallis |
| K09888: hypothetical protein\|unclassified | 7.260504 | 0.0265 | Kruskal-Wallis |
| K09893: hypothetical protein | 7.623529 | 0.0221 | Kruskal-Wallis |
| K09955: hypothetical protein\|unclassified | 6.005602 | 0.0496 | Kruskal-Wallis |
| K10112: multiple sugar transport system ATP-binding protein | 7.2 | 0.0273 | Kruskal-Wallis |
| K10201: N-acetylglucosamine transport system permease protein | 6.72 | 0.0347 | Kruskal-Wallis |
| K10201: N-acetylglucosamine transport system permease protein\|unclassified | 6.72 | 0.0347 | Kruskal-Wallis |
| K10206: LL-diaminopimelate aminotransferase [EC:2.6.1.83]\|unclassified | 6.488889 | 0.039 | Kruskal-Wallis |
| K10254: myosin-crossreactive antigen | 7.2 | 0.0273 | Kruskal-Wallis |
| K10254: myosin-crossreactive antigen\|unclassified | 7.2 | 0.0273 | Kruskal-Wallis |
| K10439: ribose transport system substrate-binding protein | 6.488889 | 0.039 | Kruskal-Wallis |
| K11069: spermidine/putrescine transport system substrate-binding protein | 7.260504 | 0.0265 | Kruskal-Wallis |
| K11069: spermidine/putrescine transport system substrate-binding protein\|unclassified | 6.72 | 0.0347 | Kruskal-Wallis |
| K11070: spermidine/putrescine transport system permease protein\|unclassified | 6.16092 | 0.0459 | Kruskal-Wallis |
| K11071: spermidine/putrescine transport system permease protein\|unclassified | 7.2 | 0.0273 | Kruskal-Wallis |
| K11072: spermidine/putrescine transport system ATP-binding protein [EC:3.6.3.31]\|unclassified | 7.2 | 0.0273 | Kruskal-Wallis |
| K11144: primosomal protein DnaI | 6.72 | 0.0347 | Kruskal-Wallis |
| K11145: ribonuclease III family protein | 7.2 | 0.0273 | Kruskal-Wallis |
| K11179: tRNA 2-thiouridine synthesizing protein E [EC:2.8.1.-]\|unclassified | 6.16092 | 0.0459 | Kruskal-Wallis |
| K11206: trypanothione amidase [EC3.5.1.-] | 7.623529 | 0.0221 | Kruskal-Wallis |
| K11753: riboflavin kinase / FMN adenylyltransferase [EC:2.7.1.26 2.7.7.2]\|unclassified | 6.72 | 0.0347 | Kruskal-Wallis |
| K11927: ATP-dependent RNA helicase RhlE [EC:3.6.4.13] | 6.005602 | 0.0496 | Kruskal-Wallis |
| K12056: conjugal transfer mating pair stabilization protein TraG | 7.623529 | 0.0221 | Kruskal-Wallis |
| K12058: conjugal transfer mating pair stabilization protein TraN | 7.623529 | 0.0221 | Kruskal-Wallis |
| K12061: conjugal transfer pilus assembly protein TraW | 7.623529 | 0.0221 | Kruskal-Wallis |
| K12063: conjugal transfer ATP-binding protein TraC | 7.623529 | 0.0221 | Kruskal-Wallis |
| K12308: beta-galactosidase [EC:3.2.1.23]\|unclassified | 6.16092 | 0.0459 | Kruskal-Wallis |
| K12506: 2-C-methyl-D-erythritol 4-phosphate cytidylyltransferase / 2-C-methyl-D-erythritol 2,4-cyclodiphosphate synthase [EC:2.7.7.60 4.6.1.12] | 6.72 | 0.0347 | Kruskal-Wallis |
| K12506: 2-C-methyl-D-erythritol 4-phosphate cytidylyltransferase / 2-C-methyl-D-erythritol 2,4-cyclodiphosphate synthase [EC:2.7.7.60 4.6.1.12]\|unclassified | 7.623529 | 0.0221 | Kruskal-Wallis |
| K12542: membrane fusion protein LapC | 7.448276 | 0.0241 | Kruskal-Wallis |
| K12542: membrane fusion protein LapC\|unclassified | 7.448276 | 0.0241 | Kruskal-Wallis |
| K12860: pre-mRNA-splicing factor CDC5/CEF1 | 7.623529 | 0.0221 | Kruskal-Wallis |
| K12860: pre-mRNA-splicing factor CDC5/CEF1\|unclassified | 7.623529 | 0.0221 | Kruskal-Wallis |
| K12940: NO_NAME | 7.448276 | 0.0241 | Kruskal-Wallis |
| K12940: NO_NAME\|unclassified | 7.623529 | 0.0221 | Kruskal-Wallis |
| K12952: NO_NAME\|unclassified | 6.72 | 0.0347 | Kruskal-Wallis |
| K13040: two-component system, LuxR family, sensor histidine kinase TtrS [EC:2.7.13.3] | 7.623529 | 0.0221 | Kruskal-Wallis |
| K13280: signal peptidase, endoplasmic reticulum-type [EC:3.4.-.-]\|unclassified | 6.72 | 0.0347 | Kruskal-Wallis |
| K13653: NO_NAME\|unclassified | 7.623529 | 0.0221 | Kruskal-Wallis |
| K13928: NO_NAME | 7.623529 | 0.0221 | Kruskal-Wallis |
| K13929: NO_NAME | 7.623529 | 0.0221 | Kruskal-Wallis |
| K13933: NO_NAME | 7.623529 | 0.0221 | Kruskal-Wallis |
| K14088: NO_NAME | 6.72 | 0.0347 | Kruskal-Wallis |
| K14088: NO_NAME\|unclassified | 6.72 | 0.0347 | Kruskal-Wallis |
| K14267: N-succinyldiaminopimelate aminotransferase [EC:2.6.1.17] | 7.623529 | 0.0221 | Kruskal-Wallis |
| K14540: NO_NAME\|unclassified | 6.763636 | 0.034 | Kruskal-Wallis |
| K14654: 2,5-diamino-6-(ribosylamino)-4(3H)-pyrimidinone 5'-phosphate reductase [EC:1.1.1.302] | 6.488889 | 0.039 | Kruskal-Wallis |
| K14654: 2,5-diamino-6-(ribosylamino)-4(3H)-pyrimidinone 5'-phosphate reductase [EC:1.1.1.302]\|unclassified | 6.488889 | 0.039 | Kruskal-Wallis |
| K14665: NO_NAME | 7.623529 | 0.0221 | Kruskal-Wallis |
| K14731: epsilon-lactone hydrolase [EC:3.1.1.83] | 7.623529 | 0.0221 | Kruskal-Wallis |
| K14731: epsilon-lactone hydrolase [EC:3.1.1.83]\|unclassified | 7.623529 | 0.0221 | Kruskal-Wallis |
| K15555: sulfonate transport system ATP-binding protein [EC:3.6.3.-] | 6.72 | 0.0347 | Kruskal-Wallis |
| K15580: oligopeptide transport system substrate-binding protein\|unclassified | 6.72 | 0.0347 | Kruskal-Wallis |
| K15583: oligopeptide transport system ATP-binding protein\|unclassified | 6.488889 | 0.039 | Kruskal-Wallis |
| K15921: arabinoxylan arabinofuranohydrolase [EC:3.2.1.55] | 7.623529 | 0.0221 | Kruskal-Wallis |
| K15921: arabinoxylan arabinofuranohydrolase [EC:3.2.1.55]\|unclassified | 7.623529 | 0.0221 | Kruskal-Wallis |
| K16087: NO_NAME | 7.623529 | 0.0221 | Kruskal-Wallis |
| K16153: phosphorylase / glycogen(starch) synthase [EC:2.4.1.1 2.4.1.11] | 6.543417 | 0.0379 | Kruskal-Wallis |
| K16153: phosphorylase / glycogen(starch) synthase [EC:2.4.1.1 2.4.1.11]\|unclassified | 6.543417 | 0.0379 | Kruskal-Wallis |
| K16898: NO_NAME\|unclassified | 6.72 | 0.0347 | Kruskal-Wallis |
| K16899: NO_NAME\|unclassified | 6.72 | 0.0347 | Kruskal-Wallis |
| K16957: L-cystine transport system substrate-binding protein | 7.623529 | 0.0221 | Kruskal-Wallis |
| K17680: NO_NAME | 7.623529 | 0.0221 | Kruskal-Wallis |
| K17810: NO_NAME\|unclassified | 7.623529 | 0.0221 | Kruskal-Wallis |
| K18014: 3-aminobutyryl-CoA ammonia-lyase [EC:4.3.1.14] | 6.72 | 0.0347 | Kruskal-Wallis |
| K18014: 3-aminobutyryl-CoA ammonia-lyase [EC:4.3.1.14]\|unclassified | 6.72 | 0.0347 | Kruskal-Wallis |
| K18138: multidrug efflux pump | 7.260504 | 0.0265 | Kruskal-Wallis |
| K18221: NO_NAME | 7.623529 | 0.0221 | Kruskal-Wallis |
| K18672: NO_NAME | 6.72 | 0.0347 | Kruskal-Wallis |
| K18672: NO_NAME\|unclassified | 6.72 | 0.0347 | Kruskal-Wallis |
| K18890: ATP-binding cassette, subfamily B, multidrug efflux pump | 7.448276 | 0.0241 | Kruskal-Wallis |
| K18890: ATP-binding cassette, subfamily B, multidrug efflux pump\|unclassified | 6.72 | 0.0347 | Kruskal-Wallis |
| K18907: NO_NAME | 7.623529 | 0.0221 | Kruskal-Wallis |
| K19005: lipoteichoic acid synthase [EC:2.7.8.20]\|unclassified | 7.623529 | 0.0221 | Kruskal-Wallis |
| K19157: NO_NAME | 7.2 | 0.0273 | Kruskal-Wallis |
| K19165: NO_NAME | 6.543417 | 0.0379 | Kruskal-Wallis |
| K19165: NO_NAME\|unclassified | 6.543417 | 0.0379 | Kruskal-Wallis |
| K19200: isopenicillin-N N-acyltransferase like protein | 7.623529 | 0.0221 | Kruskal-Wallis |
| K19271: NO_NAME | 7.623529 | 0.0221 | Kruskal-Wallis |
| K19294: NO_NAME\|unclassified | 6.72 | 0.0347 | Kruskal-Wallis |
| K19340: Cu-processing system ATP-binding protein | 7.623529 | 0.0221 | Kruskal-Wallis |
| K19341: Cu-processing system permease protein | 7.623529 | 0.0221 | Kruskal-Wallis |
| K19342: NO_NAME | 7.623529 | 0.0221 | Kruskal-Wallis |
| K20074: NO_NAME\|unclassified | 6.005602 | 0.0496 | Kruskal-Wallis |
| K20116: PTS system, glucose-specific IIA component [EC:2.7.1.199] | 6.72 | 0.0347 | Kruskal-Wallis |
| K20116: PTS system, glucose-specific IIA component [EC:2.7.1.199]\|unclassified | 6.72 | 0.0347 | Kruskal-Wallis |
| K20117: PTS system, glucose-specific IIB component [EC:2.7.1.199] | 6.72 | 0.0347 | Kruskal-Wallis |
| K20117: PTS system, glucose-specific IIB component [EC:2.7.1.199]\|unclassified | 6.72 | 0.0347 | Kruskal-Wallis |
| K20118: PTS system, glucose-specific IIC component | 6.72 | 0.0347 | Kruskal-Wallis |
| K20118: PTS system, glucose-specific IIC component\|unclassified | 6.72 | 0.0347 | Kruskal-Wallis |
| K20488: two-component system, OmpR family, lantibiotic biosynthesis response regulator NisR/SpaR\|unclassified | 6.488889 | 0.039 | Kruskal-Wallis |
| K20885: NO_NAME | 7.623529 | 0.0221 | Kruskal-Wallis |
| K21345: NO_NAME | 7.623529 | 0.0221 | Kruskal-Wallis |
| K21514: NO_NAME | 7.623529 | 0.0221 | Kruskal-Wallis |
| K21514: NO_NAME\|unclassified | 7.623529 | 0.0221 | Kruskal-Wallis |
| K21672: NO_NAME\|unclassified | 7.623529 | 0.0221 | Kruskal-Wallis |
| K21739: NO_NAME | 7.623529 | 0.0221 | Kruskal-Wallis |
| K21936: NO_NAME | 7.623529 | 0.0221 | Kruskal-Wallis |
| K22230: NO_NAME\|unclassified | 7.623529 | 0.0221 | Kruskal-Wallis |
| K22292: NO_NAME | 7.623529 | 0.0221 | Kruskal-Wallis |
| K22431: NO_NAME | 7.260504 | 0.0265 | Kruskal-Wallis |
| K22431: NO_NAME\|unclassified | 7.260504 | 0.0265 | Kruskal-Wallis |
| K22468: NO_NAME | 6.763636 | 0.034 | Kruskal-Wallis |
| K22900: NO_NAME\|unclassified | 6.763636 | 0.034 | Kruskal-Wallis |
| K22927: NO_NAME\|unclassified | 6.72 | 0.0347 | Kruskal-Wallis |
| K22958: NO_NAME | 7.623529 | 0.0221 | Kruskal-Wallis |
| K22958: NO_NAME\|unclassified | 7.623529 | 0.0221 | Kruskal-Wallis |

Table S3. Disease activity index (DAI) scoring criteria

| Score | Weight Loss (%) | Stool Consistency | Occulted Blood |
| --- | --- | --- | --- |
| 0 | ＜1 | Normal | None |
| 1 | 1-5 | Soft (Formed) | 1 |
| 2 | 6-10 | Soft (Unformed), Semi-Loose | 2 |
| 3 | 10-15 | Very Soft, Loose Stool | 3 |
| 4 | ＞15 | Loose Stool | 4 |

Note: The scoring for the fecal occult blood test should be performed according to the instructions provided in the assay kit.

Table S4. Histological evaluation scoring criteria

| Score | Pithelial/  crypt Damage | Inflammation involvement | Ulcer number |
| --- | --- | --- | --- |
| 0 | None | None | 0 |
| 1 | Goblet cells loss | Crypt | 1 |
| 2 | Goblet cells loss (Massive) | Mucosa | 2 |
| 3 | Crypt Loss | Mucosa and Submucosa | 3 |
| 4 | Crypt and Surface Epithelial Destruction | Transmural | ＞3 |

Table S5. Primer sequences used for gene expression analysis using RT-qPCR

| *Genes* | Sequences |
| --- | --- |
| *ZO-1-F* | ACCCGAAACTGATGCTGTGGATAG |
| *ZO-1-R* | AAATGGCCGGGCAGAACTTGTGTA |
| *Occludin-F* | GGACCCTGACCACTATGAAACAGACTAC |
| *Occludin-R* | ATAGGTGGATATTCCCTGACCCAGTC |
| *MUC2-F* | GCTCTCAAATGGTGCAGAGG |
| *MUC2-R* | AGTGGGAAGGGATGAATGGG |
| *Il-1β-F* | CAACCAACAAGTGATATTCTCCATG |
| *Il-1β-R* | GATCCACACTCTCCAGCTGCA |
| *Il-6-F* | CACTTCACAAGTCGGAGGCT |
| *Il-6-R* | CTGCAAGTGCATCATCGTTGT |
| *Il-10F* | AGTGGAGCAGGTGAAGAGTG |
| *Il-10R* | TTCGGAGAGAGGTACAAACG |
| *TNF-α-F* | CCTGTAGCCCACGTCGTAG |
| *TNF-α-R* | GGGAGTAGACAAGGTACAACCC |
| *TLR4-F* | GCTCTCAGCCATCCACAAAG |
| *TLR4-R* | GAGTCGGGAAGAGGAAGAGG |
| *Myd88-F* | TGCTAGAGCTGCTGGCCTTGT |
| *Myd88-R* | GGCTTCTCGGACTCCTGGTTCT |
| *Gpr41-F* | CGACTAGAGATGGCTGTGGT |
| *Gpr41-R* | AGAAGATGAGCAGTGTGGCT |
| *Gpr43-F* | TCCAGCCTGGCTTTCCAATA |
| *Gpr43-R* | GCCTGCAGGAGACATTTCAG |
| *Pparγ-F* | ATTGAGTGCCGAGTCTGTGG |
| *Pparγ-R* | GGCATTGTGAGACATCCCCA |
| *Nos2-F* | GCCCAGGAGGAGAGAGAT |
| *Nos2-R* | GCAAAGAGGACTGTGGCT |
| *18S-F* | GTAACCCGTTGAACCCCATT |
| *18S-R* | CCATCCAATCGGTAGTAGCG |

**III. Supplementary Texts**

Text S1. *In vivo* distribution of apiin and apigenin

Thirty mice were randomly assigned to two groups (n = 15) and orally administered apiin (200 mg/kg). At 0, 2, 4, 8, and 16 h post-dosing, three mice from each group were sacrificed at each time point. Serum, gastric contents, intestinal contents, ileal contents, cecal contents, colonic contents, and colonic tissues were collected for HPLC quantitation. Apiin and apigenin were extracted using pre-cooled methanol-water (v:v = 4:1), concentrated under a gentle stream of nitrogen, and redissolved in methanol-water (v:v = 1:4).

Text S2. HPLC analysis of apiin and apigenin

HPLC analysis was performed on a Shimadzu LC-20A system (Shimadzu Scientific Instruments, Kyoto, Japan), equipped with a SHIMSEN Superb II C18 column (4.6 × 250 mm, 5 µm). The mobile phase consisted of acetonitrile:water (5:5 v/v) and methanol; detection wavelength was 210 nm. Standard curves for apiin (0.001~0.5 mg/mL) and apigenin (0.001~1 mg/mL) were generated in a good linear relationship (R² ≥ 0.995). The concentrations of apiin- or apigenin-containing samples were analyzed in this HPLC system.

Text S3. NMR analysis

Apiin and apigenin (5 mg each) were dissolved in 0.6 mL deuterated DMSO containing 0.03% TMS. 1H-NMR spectra were acquired using an AVANCE III HD 400 MHz spectrometer (Bruker Corporation, Billerica, MA, USA). Spectra were recorded and analyzed using MestReNova 14.0.
